# Tobacco plants expressing the maize nitrate transporter ZmNrt2.1 exhibit altered responses of growth and gene expression to nitrate and calcium

**DOI:** 10.1186/s40529-017-0203-9

**Published:** 2017-11-15

**Authors:** Abubakar Ibrahim, Xiao-Lu Jin, Yu-Bin Zhang, Jessica Cruz, Paveena Vichyavichien, Nwadiuto Esiobu, Xing-Hai Zhang

**Affiliations:** 1 Department of Biological Sciences, Florida Atlantic University, Boca Raton, FL 33431 USA; 2 Department of Soil Sciences, Modibbo Adama University of Technology, Yola, Nigeria; 3 Monitoring Center for Marine Resources and Environments, Guangdong Ocean University, Zhanjiang, 524088 China

**Keywords:** Calcium signaling, Maize high-affinity nitrate transporter ZmNrt2.1, Nitrate sensing, Nitrate responsive genes, Potassium, Tobacco

## Abstract

**Background:**

Nitrate uptake is a highly regulated process. Understanding the intricate interactions between nitrate availability and genetically-controlled nitrate acquisition and metabolism is essential for improving nitrogen use efficiency and increasing nitrate uptake capacity for plants grown in both nitrate-poor and nitrate-enriched environments. In this report, we introduced into tobacco (*Nicotiana tabacum*) the constitutively expressed maize high-affinity transporter ZmNrt2.1 gene that would bypass the tight control for the endogenous nitrate-responsive genes. By using calcium inhibitors and varying levels of NO_3_^−^, Ca^2+^ and K^+^, we probed how the host plants were affected in their nitrate response.

**Results:**

We found that the ZmNrt2.1-expressing plants had better root growth than the wild type plants when Ca^2+^ was deficient regardless of the nitrate levels. The growth restriction associated with Ca^2+^-deficiency can be alleviated with a high level of K^+^. Furthermore, the transgenic plants exhibited altered expression patterns of several endogenous, nitrate-responsive genes, including the high- and low-affinity nitrate transporters, the Bric-a-Brac/Tramtrack/Broad protein BT2 and the transcription factor TGA-binding protein TGA1, in responding to treatments of NO_3_^−^, K^+^ or inhibitors for the calcium channel and the cytosolic Ca^2+^-regulating phospholipase C, as compared to the wild type plants under the same treatments. Their expression was not only responsive to nitrate, but also affected by Ca^2+^. There were also different patterns of gene expression between roots and shoots.

**Conclusion:**

Our results demonstrate the ectopic effect of the maize nitrate transporter on the host plant’s overall gene expression of nitrate sensing system, and further highlight the involvement of calcium in nitrate sensing in tobacco plants.

**Electronic supplementary material:**

The online version of this article (10.1186/s40529-017-0203-9) contains supplementary material, which is available to authorized users.

## Background

Nitrogen is an essential element for all forms of life. Nitrate (NO_3_^−^) is a major form of nitrogen in the environment taken up by plants (Guerrero et al. 1981). Because of high demand for nitrate by crop plants to achieve high yield, over 100 million tons of nitrogen-fertilizers are being applied annually to farm land worldwide in recent years (FAO 2015). Yet due to low affinity between nitrate and most types of soils, as much as 50–70% of the applied nitrogen may be leached from the soil (Peoples et al. 1995), causing pollution and eutrophication of natural water systems, especially near agricultural and urban areas (Unkles et al. 2004; Howarth and Marino 2006). Better understanding of how plants respond to nitrate availability in soil and regulate nitrate uptake is important in finding suitable solutions to over-fertilization as well as remedy for nitrogen deficiency.

Plants take up NO_3_^−^ from soil by transporting it across the plasma membrane of the root epidermal and cortical cells. This process is carried out by various nitrate transporters through a proton-coupled gradient 2H^+^/1NO_3_^−^ symport mechanism (Meharg and Blatt 1995; Crawford and Glass 1998). Once inside the cell, most nitrate is first reduced to nitrite by nitrate reductases, and then to ammonium by nitrite reductases. Ammonium is metabolized into glutamine, a universal precursor for many metabolic processes (Guerrero et al. 1981; Good et al. 2004; Wang et al. 2012). Plants have developed multiple and independently operating systems to adjust for changing nitrate availability in the environment. There are at least three known nitrate uptake systems: a constitutive high-affinity transport system (cHATS), a NO_3_^−^ inducible high-affinity transport system (iHATS), and a low-affinity constitutively expressed transport system (LATS) (Crawford and Glass 1998; Forde 2000). Previous studies have shown that the LATS operates when NO_3_^−^ levels are high (> 1 mM), whereas the cHATS and iHATS operate at lower NO_3_^−^ levels (< 1 mM) (Crawford and Glass 1998; Quaggiotti et al. 2003; Katayama et al. 2009). Although the cHATS has a higher affinity for NO_3_^−^, the iHATS is more active with a V_max_ 30 times that of cHATS and is responsible for a greater NO_3_^−^ uptake (Kronzucker et al. 1996; Crawford and Glass 1998). Upon exposed to nitrate, nitrogen starved plants increase nitrate uptake several folds largely due to the iHATS (Tsay et al. 2007).

In Arabidopsis, at least four families of nitrate-transporting proteins have been studied: low-affinity nitrate transporter 1/peptide transporter (NRT1 or NPF), high-affinity nitrate transporter 2 (NRT2), chloride channel and slow anion channel associated homologues (Forde 2000; Quaggiotti et al. 2003; Krapp et al. 2014). Members of NRT1 family have been shown to play a variety of important physiological roles such as sustaining NO_3_^−^ nitrate uptake and influx, transporting plant hormones, regulating NRT2 gene expression and HATS activity, and nitrate and calcium sensing (Muños et al. 2004; Remans et al. 2006; Chiba et al. 2015; Undurraga et al. 2017). Interestingly, unlike NRT2, NRT1.1 gene is not induced by nitrate deficiency nor suppressed by reduced nitrogen metabolism (Lejay et al. 1999). But Loqué et al. (2003) reported repression of NTR1.1 gene expression by the reduced NO_3_^−^ product NO_2_^−^ in Arabidopsis roots.

The NRT2 family is a part of the iHATS responsible for the influx of nitrate into the plant cell when nitrate becomes available in the environment. For example, the Arabidopsis NRT2.1 is crucial for NO_3_^−^ influx especially when nitrate is deficient (Wirth et al. 2007). Structurally, NRT2 nitrate transporters belong to the nitrate-nitrite transporter family, a part of the Major Facilitator Superfamily of membrane transporters (Pao et al. 1998). Thus, the NRT2 membrane topology is made of two sets of six transmembrane domains connected by a cytosolic loop (Pao et al. 1998; Forde 2000). For several plants investigated, the transport activity of NRT2 protein is shown to require the direct interaction with another protein, NAR2 (Quesada et al. 1994; Zhou et al. 2000; Orsel et al. 2006). For example, in Arabidopsis both the NRT2.1 and NAR2.1 proteins were shown to localize and interact at the plasma membrane in the roots, where the active nitrate transporter is presumably formed as a NRT2.1/NAR2.1 heterooligomer (Wirth et al. 2007; Yong et al. 2010). In a study carried out in barley, nitrate and nitrite transport activity was only observed when HvNRT2.1 and HvNAR2.3 genes were both injected into the *Xenopus* oocytes expression system, and no transport activity was detected when HvNRT2.1 gene was injected alone or coupled with HvNAR2.1 or HvNAR2.2 genes (Tong et al. 2005). In maize roots, the accessory protein ZmNAR2.1 (ZmNRT3.1A) was important for iHATS activity and appeared to form an oligomer with ZmNRT2.1 (Pii et al. 2016). Although the two-component system in several species tested seems to be specific for NRT and NAR proteins, the rice OsNAR2.1 had a broader range of interactions with OsNRT2.1, OsNRT2.2 and OsNRT2.3a (Yan et al. 2011). Furthermore, the NRT2 genes in grass species are distinct from the Arabidopsis NRT2 by the absence of gene family clusters in the genomes and the lack of an intron, suggesting that the divergence of the NRT2 family occurred after the evolutionary split between dicots and monocots (Plett et al. 2010).

The iHATS including NRT2 is part of nitrate sensing system tightly controlled to maintain nitrogen homeostasis, where its activity dramatically increases upon first provision of NO_3_^−^ and is quickly repressed after NO_3_^−^ exposure (Crawford and Glass 1998; Quaggiotti et al. 2003; Medici and Krouk 2014). Down-regulation occurs through mRNA stability and with influx of other nitrogen metabolites such as ammonium, glutamate, glutamine, asparagine, and arginine (Imsande and Touraine 1994; Forde and Clarkson 1999). Growth and development is another signal for NRT2 regulation. For example, in Arabidopsis NRT2.1 protein levels remain stable in older plants and are not affected by environmental cues such as nutrient availability or darkness, while in younger (8-day old) seedlings the amount of NRT2.1 protein is decreased after 24 h of darkness (Laugier et al. 2012). Another study showed that light, sucrose or nitrogen treatments strongly affect both NRT2.1 transcription and HATS activity in Arabidopsis, but NRT2.1 protein level remains largely steady in response to these treatments (Wirth et al. 2007). Yet a different study reported that cellular glucose elevates NRT2.1 protein levels and transport activity in Arabidopsis, independent of NRT2.1 transcription (de Jong et al. 2014). Furthermore, posttranscriptional control was reported to be important for NRT2.1/NAR2.1 transport system in Arabidopsis roots (Laugier et al. 2012) and NRT2.1-nitrate influx in *Nicotiana plumbaginifolia* (Fraisier et al. 2000). Regardless of the various points of view and some seemingly discrepancies in literature, it is clear that NRT2.1 is actively regulated at various levels of transcription and translation, and there is an intricate crosstalk between plant metabolism and nitrate gene expression throughout growth and development.

In plants, hundreds of genes, including the aforementioned NO_3_^−^ uptake systems and nitrate transporters (NRT), respond to nitrate as a regulatory signal (Wang et al. 2004; Krapp et al. 2014; Medici and Krouk 2014). However, increasing evidence has shown that calcium is another essential player in the nitrate signaling network. For example, Ca^2+^ and calcium-binding proteins such as CIPKs are important in modulating NRT gene expression in response to cellular and environmental nitrate levels (Albrecht et al. 2001; Hu et al. 2009). The universal calcium-mobilizing second messenger, inositol 1,4,5-trisphosphate, is produced by phosphoinositide-specific phospholipase C (PLC) enzymes from hydrolyzing the highly phosphorylated lipid phosphatidylinositol 4,5-bisphosphate (Streb et al. 1983; Hunt et al. 2004). Changes in cellular Ca^2+^ levels through the actions of PLC and membrane-bound calcium-permeable channels can significantly affect the expression of nitrate-responding genes (Sakakibara et al. 1997; Riveras et al. 2015). Thus, inhibitors such as U73122 for PLC (Franklin-Tong et al. 1996) and La^3+^ for several calcium channels (White 2000) have become a useful tool for analyzing Ca^2+^- or nitrate-responsive genes.

It is obvious that nitrate uptake and metabolism in plants is tightly regulated by various signals at different levels (Wang et al. 2012; Krapp et al. 2014; Medici and Krouk 2014). Studies of the high-affinity nitrate transporter NRT2, a major nitrate uptake avenue for plants, and other nitrate responsive and regulatory genes will help better understand the intricate interactions between nitrate availability in the environment and genetically-controlled nitrate acquisition and metabolism. This knowledge is needed for achieving high nitrogen use efficiency and high capacity of nitrate uptake for plants in both nitrate-poor and anthropologically nitrate-enriched environments, in order to aim for an optimal balance between fertilizer usage, plant productivity and environmental protection (Good et al. 2004). As part of the effort to investigate plant nitrate response and regulation, we introduced in tobacco plants a maize high-affinity transporter ZmNrt2.1 gene driven by the constitutive CaMV 35S promoter. Therefore, this ZmNrt2.1 transgene would bypass the tight transcriptional regulation exerted on host plant’s endogenous nitrate-responsive genes. Thus, we hoped to use ZmNrt2.1, along with calcium inhibitors and varying availability of NO_3_^−^, Ca^2+^ and K^+^, to probe changes of nitrate responses in the host plant. Our studies reveal the ectopic effect of ZmNrt2.1 on growth and nitrate sensing system involving several endogenous nitrate-responsive genes.

## Materials and methods

### Plant material

As described previously (Hill et al. 2016), plants of tobacco (*Nicotiana tabacum* L., cv. Havana petit) were grown in MS medium solidified with 2.7 g/L phytagel in a growth chamber at 24°C under 8 h of darkness and 16 h of cool white light of 150 μmol photons m^−2^ s^−1^. The seedlings were used for various treatments as described below.

### Construction of transformation vector expressing maize nitrate transporter

The coding sequence of the high-affinity nitrate transporter gene ZmNrt2.1 from maize (*Zea mays*, Genbank accession: NM_001111725; Quaggiotti et al. 2003), fused with the FLAG tag (DYKDDDDK) coding sequence, was synthesized by GenScript (USA). The 1.6-kb *Sma*I/*Sac*I-cut fragment was ligated into the *Sma*I/*Sac*I digested binary plasmid E1492 (Zhang et al. 2004) that is derived from pBI101 (Clontech, USA). The resultant plasmid p35S-ZmNrt (Additional file 1: Figure S1) was introduced to *Agrobacterium tumefaciens* strain EHA105.

### *Agrobacterium*-mediated plant transformation

Plant transformation was carried out as previously described (Zhang et al. 2004; Tsai et al. 2010; Hill et al. 2016) using kanamycin (100 mg/L) as selection reagent. Eight independent, homozygous lines of transgenic plants were identified by screening T1 and T2 seeds germinated on kanamycin-containing medium. Based on the segregation ratios, plants with a single copy ZmNrt2.1 transgene were identified. Three independent lines of these plants were used for this study.

### DNA, RNA and protein analysis

Genomic DNA was extracted from leaves of the wild type and transgenic plants using Plant DNeasy kit (Qiagen, USA). Polymerase chain reaction (PCR) was carried out to confirm the presence of ZmNrt2.1 in the transgenic plants.

Total RNA was extracted from shoots or roots of the wild type and transgenic plants in similar physiological conditions or under different treatments using Qiagen Plant RNeasy kit, including a DNase treatment to remove residual DNA. The first-strand cDNA was synthesized using M-MLV Reverse Transcriptase RNase H^−^ kit (Solis BioDyne, Estonia) with random primers and oligo dT_18_. Quantitative real-time PCR (qPCR) using SYBR Green (Life Technologies, USA) was carried out to examine the transcription of the transgene ZmNrt2.1 and several endogenous genes with gene-specific primers. Tobacco elongation factor 1α and ribosomal large subunit protein L25 (rpL25) cDNAs were used as reference for qPCR, according to Schmidt and Delaney (2010). The information for all the primers used in this study is listed in Additional file 1: Table S1.

To detect the presence of the ZmNrt protein, proteins were extracted from same leaf area of the wild type and transgenic plants according to Zhang et al. (2011) and Hill et al. (2016). Western blot analysis was done as described previously (Zhang et al. 2011, 2014), using monoclonal FLAG tag antibody (Cell Signaling, USA).

### qPCR analysis of tobacco endogenous nitrate-responsive genes

To examine changes in expression of the endogenous nitrate-responsive genes in the wild type and transgenic tobacco plants, the homologous genes in tobacco were identified by analyzing the *Arabidopsis* and *Nicotiana* sequence databases. Gene specific primers (Additional file 1: Table S1) were designed for qPCR analysis of tobacco Bric-a-Brac/Tramtrack/Broad gene family member BT2 (GenBank Accessions: XM_016603770 and XM_016606879), leucine-zipper transcription factor TGA-binding protein TGA1 (KJ808740 and M62855), NAR2.1 (XM_009799779 and XM_009612602), NRT1.1 (AB102805 to AB102808) and NRT2.1 (AJ557583 and AJ557584).

### Nitrate and protein determination

NO_3_^−^ concentrations were determined from an aqueous extraction of 0.2 g frozen leaves and roots using the method described in Liu et al. (2014), spectrophotometrically measured at 410 nm using NaNO_3_ as standard. Protein extraction and measurement was done according to Hill et al. (2016).

### Nutrient treatments

Seven-day-old seedlings grown on MS medium were transferred to the medium with various NO_3_^−^, Ca^2+^ and K^+^ concentrations, and grown for 14 days in a growth chamber at 24 °C under 8 h of darkness and 16 h of light at 150 μmol photons m^−2^ s^−1^. Root length and biomass were measured. Samples of roots and shoots were also collected for gene expression assays.

Plant nutrient solutions were purchased from LaMotte (USA). The medium nutrient compositions are listed in Additional file 1: Table S2.

### Inhibitor treatments

Twenty-one-day-old seedlings of the wild type and ZmNrt2.1-expressing plants grown on MS medium were removed from the medium, gently rinsed with water and incubated with gentle shaking for 1 h in water, 5 mM LaCl_3_, 10 μM U73122 or 10 μM U73343, followed by incubation for 1 h in either 5 mM KNO_3_ or 5 mM KCl, according to Riveras et al. (2015). Roots and shoots were collected, used immediately for RNA extraction or flash frozen in liquid N_2_ and stored at – 80 °C. LaCl_3_, U73122 and U73343 were purchased from Sigma-Aldrich (USA). LaCl_3_ was dissolved in water. U73122 and U73343 were dissolved in a minimal amount of dimethyl sulfoxide and diluted in water to the final concentration. All the solutions were adjusted to pH 6.5.

### Statistical analysis

At least three replicates for each assay were performed. The differences were detected using one way ANOVA coupled with Games-Howell (equal variance not assumed) and the Least Significant Difference (LSD) methods (equal variance assumed) with the corresponding patterns of experimental data. Statistical analysis was performed using SPSS software (ver. 16.0). The significant level was set at P < 0.05.

## Results

### Generation of transgenic plants expressing the maize high-affinity nitrate transporter ZmNrt2.1

The maize high-affinity nitrate transporter ZmNrt2.1 is composed of 524 amino acids, and shares 69% residue identity and 82% similarity with the tobacco 530-amino acid NtNrt2.1 (Additional file 1: Figure S2). Kanamycin-resistant transgenic plants generated via *Agrobacterium*-mediated transformation were initially screened by PCR, an example of which is shown in Additional file 1: Figure S3. All the transgenic plants grew and reproduced normally, with no obvious phenotypic differences as compared to the untransformed wild type plants. Homozygous lines of transgenic plants were used in this study.

While RT-PCR analyses indicate an absence of ZmNrt2.1 transcription in the wild type plants, various levels of ZmNrt2.1 mRNA were detected in leaves and roots of the transgenic ZmN plants grown in the nutrient-rich MS medium (Additional file 1: Figures S4 and S5). As expected, the expression of the tobacco endogenous nitrate transporter genes NtNrt2.1 (high-affinity) and NtNrt1.1 (low-affinity) was also detected in the leaf and root of both the wild type plants and transgenic plants (Additional file 1: Figures S4 and S5). Likewise, Western blot analyses detected the presence of FLAG-fused ZmNrt2.1 protein in the transgenic plants but not in the wild type plants (Additional file 1: Figure S5). These results demonstrate that as expected, both the wild type and transgenic plants expressed the endogenous genes such as NtNrt2.1 and NtNrt1.1, and the transgenic ZmN plants also expressed the maize ZmNrt2.1. Three independent ZmN lines with a single transgene, along with the untransformed wild type plants, were studied in parallel. Similar patterns of changes and responses were observed in all these ZmNrt2.1-expressing transgenic plant lines examined. Here, we present the data on the plant line ZmN6.

### Growth response to various nitrate treatments

**Fig. 1:**
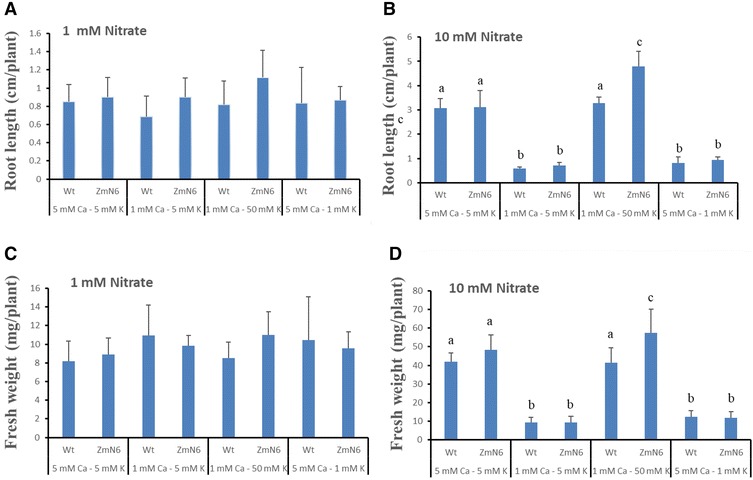
Growth of 7 days-seedlings under 1 mM nitrate (**A**, **C**) or 10 mM nitrate (**B**, **D**) and various Ca^2+^ and K^+^ concentrations for 14 days. Primary root length (**A**, **B**) and fresh biomass (**C**, **D**) of six to ten plants were measured. Wt: wild type, ZmN6: transgenic plant expressing ZmNrt2.1. Error bars indicate the standard deviation. Different letters indicate significant differences (P < 0.05) between plants-treatments of each panel, whereas no letter or same letters indicate insignificant differences

For the growth analysis, our control (“normal”) medium contained NO_3_^−^, Ca^2+^ and K^+^ at 10, 5 and 5 mM, respectively (see medium compositions in Additional file 1: Table S2). The nitrate contents of leaves or roots were largely similar between the wild type plants and the transgenic plants (Additional file 1: Figure S6). This is consistent with previous observations of similar NO_3_^−^ contents between the wild type and CaMV 35S-NpNrt2.1 transgenic *Nicotiana plumbaginifolia* plants regardless of NO_3_^−^ levels supplied (Fraisier et al. 2000). When grown under relatively low nitrate (1 mM) but normal Ca^2+^ and K^+^ conditions, both the wild type and ZmNrt2.1-expressing plants had similar primary root growth. However, under low levels (both 1 mM) of NO_3_^−^ and Ca^2+^, the transgenic ZmN6 plants grew longer primary roots than the wild type (Fig. 1A, B), which is consistent with previous reports that constitutively expressing the NRT2.1 gene is associated with increased growth (Fraisier et al. 2000; Laugier et al. 2012). On the other hand, both the wild type and ZmN6 plants grew longer primary roots under 10 mM NO_3_^−^ with normal levels of Ca^2+^ (5 mM) and K^+^ (5 mM) than when either Ca^2+^ or K^+^ was low. However, under 10 mM nitrate, Ca^2+^-restricted root growth can be compensated with a high level (50 mM) of K^+^ for both plants, and ZmN6 plants grew significantly longer primary roots than the wild type (Fig. 1A, B and Additional file 1: Figure S7).

Likewise, under 1 mM nitrate, regardless of Ca^2+^ or K^+^ levels, both the wild type and ZmN6 plants had similar biomass (Fig. 1C, D). As expected, under 10 mM nitrate both plants had higher biomass when Ca^+2^ and K^+^ were sufficient (Fig. 1D). Again, high (50 mM) K^+^ level was able to overcome Ca^2+^-deficient (but not low NO_3_^−^) caused growth restriction for both the ZmN and the wild type plants, when compared to the growth under 5 mM K^+^, and the ZmN plants grew better than the wild type (Fig. 1C, D and Additional file 1: Figure S7). Overall, these observations point to a role of Ca^2+^ and K^+^ in nitrate response and also hint at altered nitrate sensing in the ZmN plants involving both Ca^2+^ and K^+^, indicating a consequence of ectopic expression of the maize nitrate transporter gene. This prompted us to further examine expression in roots and shoots of several endogenous genes in response to NO_3_^−^, Ca^2+^ and K^+^.

### Altered responses in expression of the endogenous, nitrate-responsive genes in roots

**Fig. 2:**
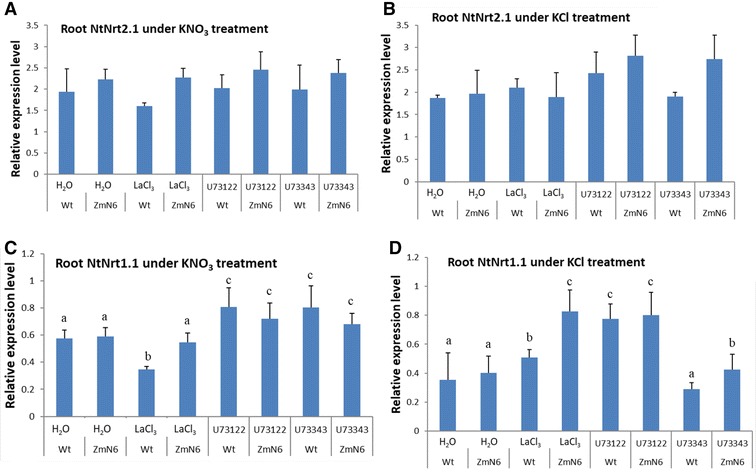
Expression of root high-affinity (NtNrt2.1; **A**, **B**) and low-affinity (NtNrt1.1; **C**, **D**) nitrate transporter genes in response to calcium inhibitor and nitrate treatments. Three-week-old plants were incubated for 1 h in H_2_O, 5 mM LaCl_3_, 10 μM U73122 or 10 μM U73343, followed by 1 h in 5 mM KNO_3_ or 5 mM KCl. The significant differences (P < 0.05) for multiple comparisons are shown with different letters separately for each panel, whereas no letter or same letters indicate insignificant variation

Under 1-h KNO_3_ treatment, the calcium channel blocker LaCl_3_ had largely no effect on the transcription of the endogenous nitrate transporter genes NtNrt2.1 and NtNrt1.1 in the roots of the ZmN transgenic plants, but LaCl_3_ inhibited these genes in the wild type plants (Fig. 2A, C). By contrast, no inhibitory effect of LaCl_3_ was observed for both plants when nitrate was absent (Fig. 2B, D). On the other hand, the phospholipase C (PLC) inhibitor U73122 that blocks increases in the cytosolic Ca^2+^ levels had no effect on NtNrt2.1 gene expression regardless of whether nitrate was present, similar as its nonfunctional analog U73343 (Fig. 2A, B). For NtNrt1.1, U73122 and U73343 treatments exhibited largely similar expression levels (Fig. 2C, D), suggesting a lack of inhibition. In stark contrast to the NtNrt2.1 gene, the transcription of NtNAR, a protein functionally associated with NtNrt2.1, was inhibited by LaCl_3_ in the transgenic ZmN roots, but not the wild type roots, in the presence of either KNO_3_ or KCl. U73122, on the other hand, variously inhibited NtNAR genes for both plants (Additional file 1: Figure S7). These results suggest that (1) calcium, nitrate and potassium all influenced the expression of these genes, and (2) the ZmNrt2.1-expressing plants showed altered responses of the native genes NtNrt2.1 and NtNAR to changes in the calcium channels and cytosolic Ca^2+^ levels, as compared to the wild type plants.

**Fig. 3:**
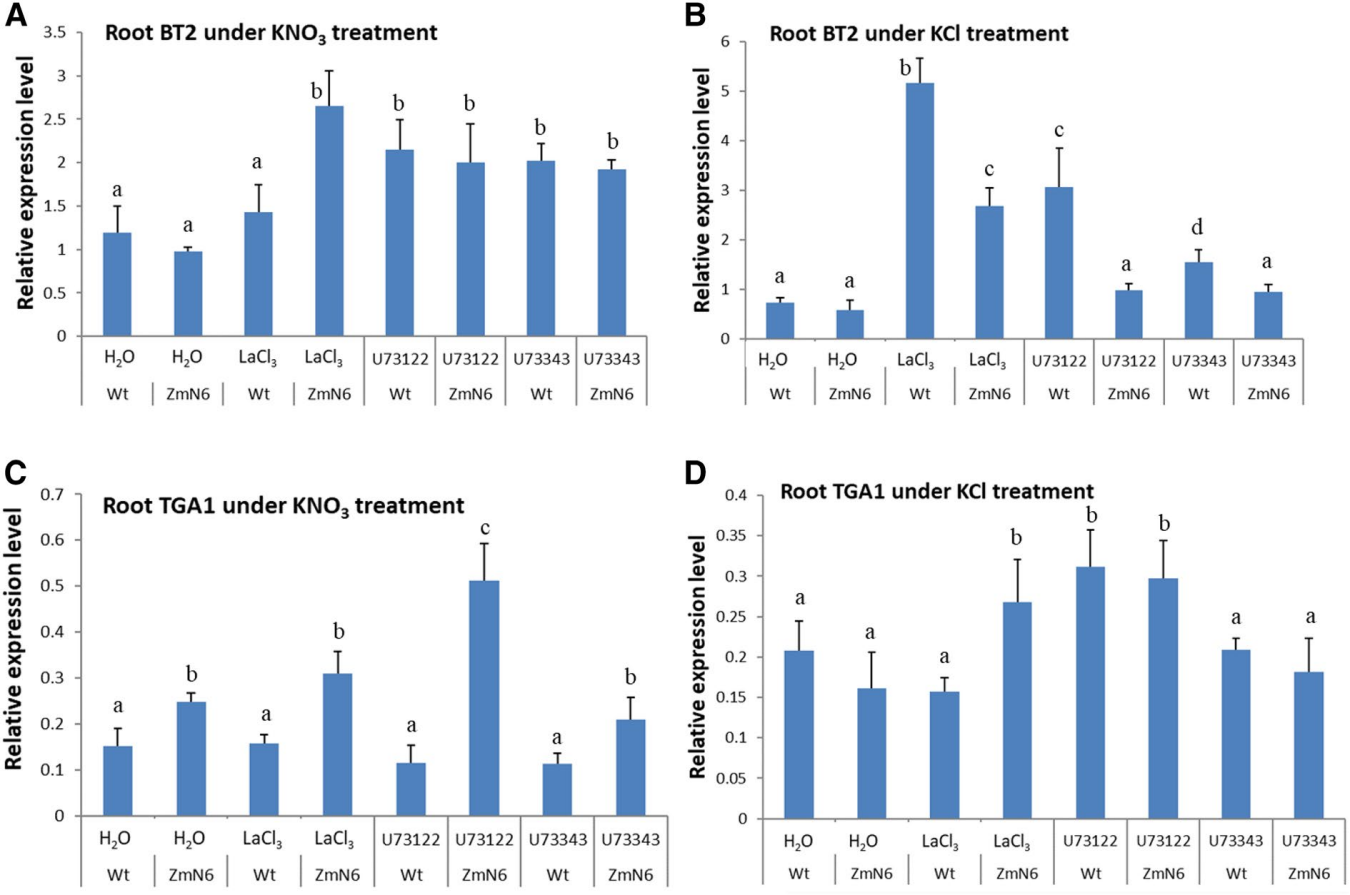
Expression of root Bric-a-Brac/Tramtrack/Broad gene BT2 (**A**, **B**) and transcription factor TGA-binding protein gene TGA1 (**C**, **D**) genes in response to calcium inhibitor and nitrate treatments, as described in Fig. 2

In Arabidopsis, a member of Bric-a-Brac/Tramtrack/Broad gene family, BT2, has been shown to be regulated by multiple signals including nitrogen nutrients and to suppress nitrate uptake (Mandadi et al. 2009; Araus et al. 2016). We examined the responses of the tobacco BT2 gene to calcium and nitrate. When treated with LaCl_3_ or U73122 followed by KNO_3_, the BT2 gene expression was elevated for both the wild type and ZmN plants (Fig. 3A), implicating a down-regulatory role by Ca channels and cytosolic [Ca^2+^]. Under KCl without nitrate, there was also a significant increase in BT2 expression with LaCl_3_ and U73122 treatments as compared to H_2_O only treatment, but the wild type exhibited a stronger response than the ZmN plants (Fig. 3B). This indicates a potassium effect on BT2 genes when nitrate was absent and calcium signaling pathway was impaired, reflecting a complex crosstalk among NO_3_^−^, Ca^2+^ and K^+^ in regulating genes for nitrate nutrition. Again, the ZmN transgenic plants exhibited altered responses different from the wild type plants.

Furthermore, the transcription factor TGA-binding protein, TGA1, is reported to be responsive to nitrate and may play a regulatory role in nitrate sensing (Ho and Tsay 2010; Alvarez et al. 2014). We found that the endogenous TGA1 expression in the ZmN roots was different from that in the wild type roots. Under KNO_3_ treatment, TGA1 transcription in the ZmN plants was increased after incubation with LaCl_3_ or U73122, whereas any enhancement of TGA1 expression was abolished by these inhibitors in the wild type plants (Fig. 3C). On the other hand, without NO_3_^−^, LaCl_3_ and U73122 did not show inhibitory effect on TGA1 transcription in both plants (Fig. 3D). This suggests that while K^+^ may not be directly involved in TGA1 gene expression, Ca^2+^ is important in mediating the nitrate-sensing TGA1 gene expression. What is revealing is that the ZmNrt2.1-expression plants under nitrate induction exhibited an altered pattern of TGA1 expression after treatment with Ca inhibitors LaCl_3_ and U73122 (Fig. 3C), again highlighting the effect of the heterologous ZmNrt2.1 gene on the overall nitrate-related gene expression in the host plant.

### Altered responses in expression of the endogenous, nitrate-responsive genes in shoots

**Fig. 4:**
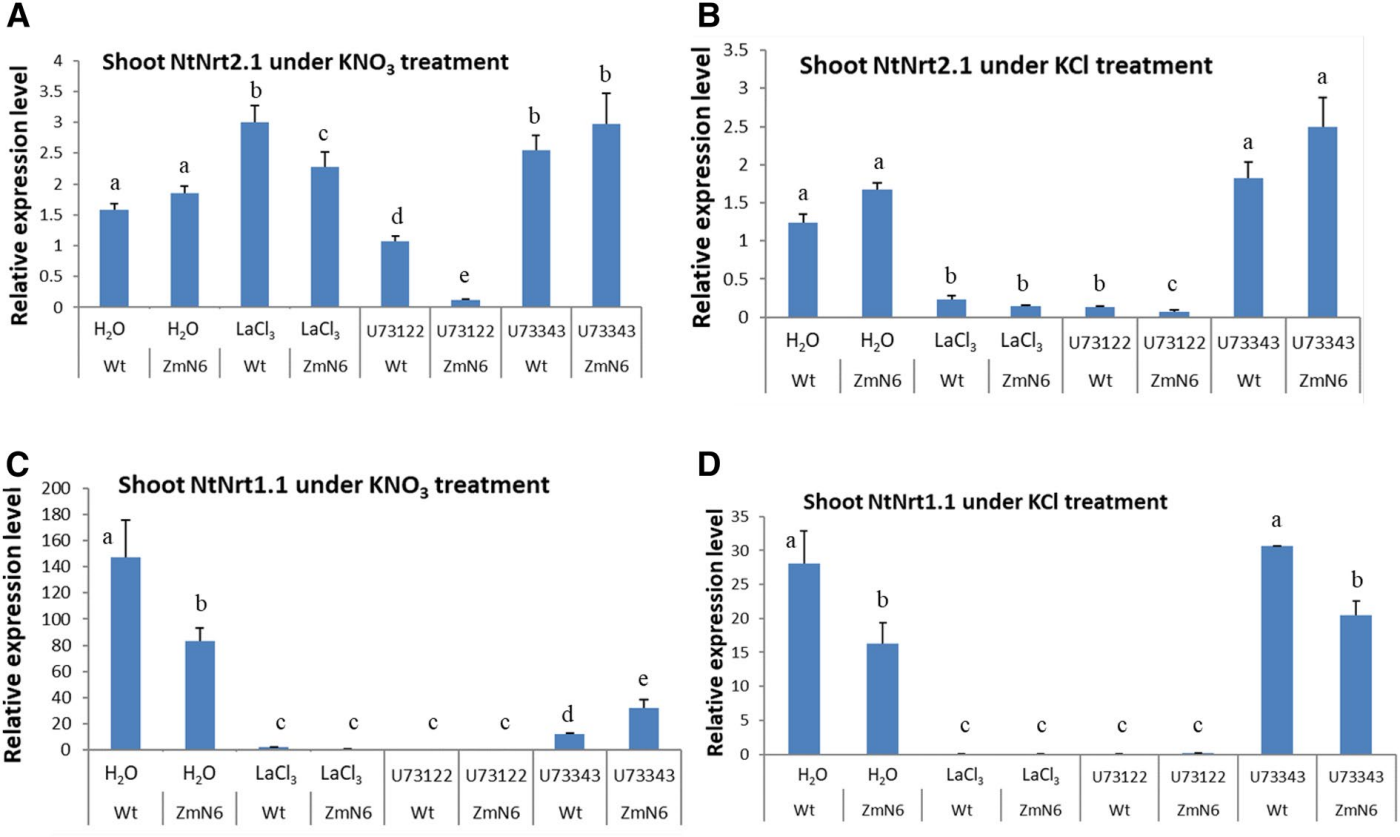
Expression of shoot nitrate transporter genes NtNrt2.1 (**A**, **B**) and NtNrt1.1 (**C**, **D**) in response to calcium inhibitor and nitrate treatments, as described in Fig. 2

Similarly, the shoots of the wild type and ZmN plants also exhibited some differences in gene expression as well as from the roots. In the presence of KNO_3_, LaCl_3_ did not inhibit NtNrt2.1 for both the wild type and ZmN plants (Fig. 4A), similar as in roots (Fig. 2A). U73122, however, almost completely abolished the NtNrt2.1transcription in the ZmN plants, whereas only partial inhibition was measured for the wild type (Fig. 4A). Without NO_3_^−^, LaCl_3_ and U73122 almost completely inhibited NtNrt2.1 expression for both plants as compared to H_2_O only treatment (Fig. 4B), which is in stark contrast to the roots where no obvious inhibition was observed (Fig. 2B). Likewise, the shoot NtNrt1.1 gene in both plants was completely inhibited by LaCl_3_ or U73122, under either KNO_3_ or KCl (Fig. 4C, D), suggesting that the calcium channel and PLC-regulated Ca^2+^ signaling play a significant role in the expression of nitrate transporter genes in shoots. These results also highlight root/shoot differences in nitrate transporter gene expression. This organ differential expression was observed for NtNAR genes as well, which in shoots exhibited almost identical patterns as NtNrt2.1 but was quite different from the roots (Fig. 4A, B; Additional file 1: Figure S8).

**Fig. 5:**
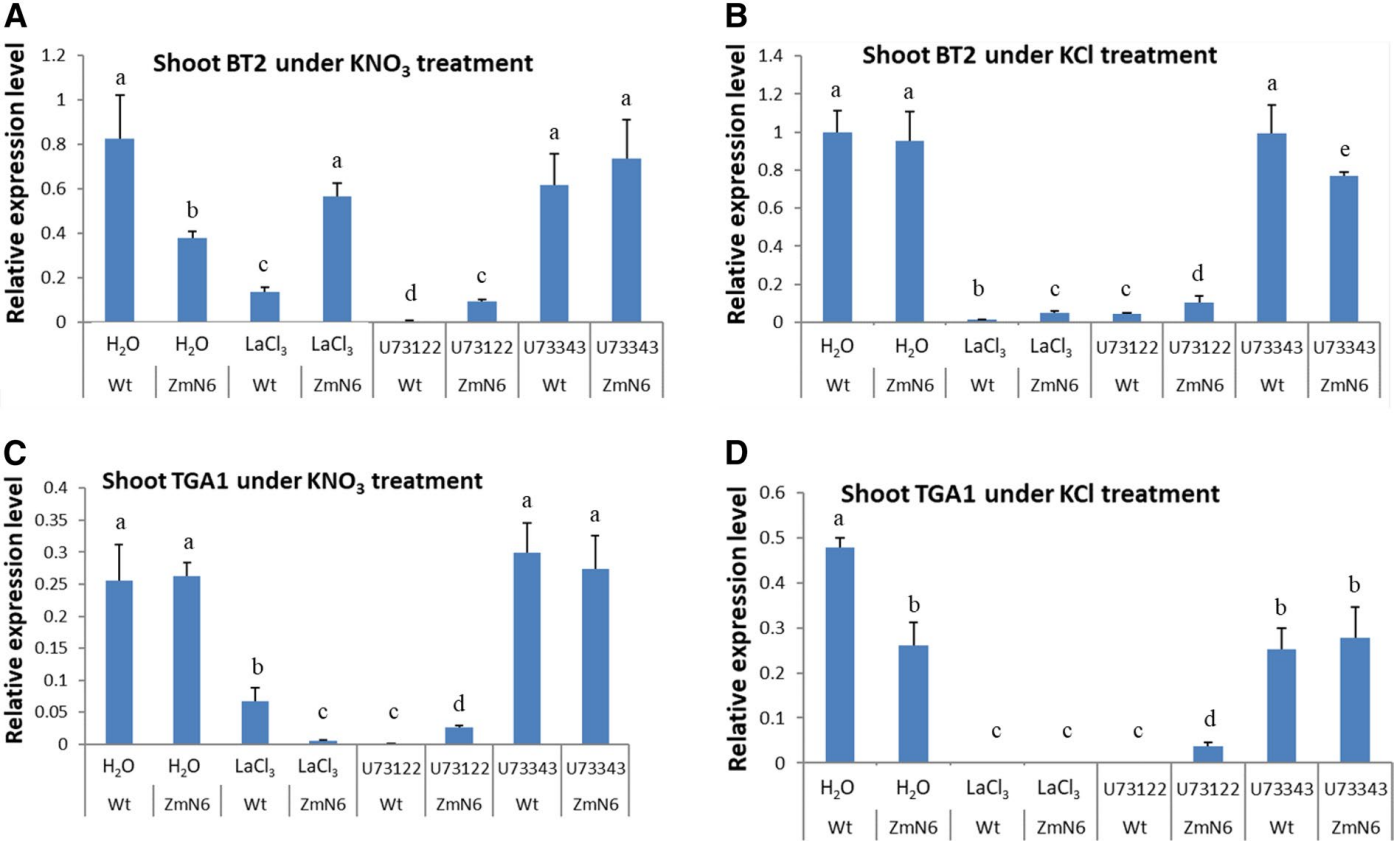
Expression of shoot BT2 (**A**, **B**) and TGA1 (**C**, **D**) genes in response to calcium inhibitor and nitrate treatments, as described in Fig. 2

Unlike in roots, BT2 gene expression in shoots of the wild type plants under KNO_3_ treatment was significantly inhibited by both LaCl_3_ and U73122, whereas for the ZmN plants this inhibition was either not observed (for LaCl_3_) or much less severe than the wild type (for U73122) (Fig. 5A). Without nitrate, on the other hand, BT2 expression was nearly completely abolished by these two inhibitors for both plants (Fig. 5B). Similarly, the TGA1 expression in shoots was significantly inhibited by both LaCl_3_ and U73122 regardless of whether KNO_3_ or KCl was present, although subtle difference can be detected between these two plants (Fig. 5C, D). All these results implicate the involvement of the Ca^2+^ signaling pathway in nitrate-related gene expression, thus nitrate sensing. Meanwhile, compared to the wild type plants, the ZmNrt2.1-expressing plants exhibited certain altered patterns of nitrate-related gene expression in response to nitrate availability and Ca^2+^ signaling inhibitors, reflecting the ectopic effect of the maize nitrate transporter on nitrate sensing in the transgenic plants.

## Discussion

A main goal for this study was to investigate what would happen to the host plant’s nitrate signaling if a foreign nitrate transporter gene, a member of the NO_3_^−^ inducible high-affinity transport system (iHATS), was introduced and expressed. Our results show that the plants expressing the maize high-affinity nitrate transporter ZmNrt2.1 had an overall better primary root growth and higher biomass than the wild type when grown under high nitrate and K^+^ but Ca^2+^ deficient conditions, whereas there was no significant difference when nitrate was deficient (Fig. 1). One explanation may be that the additional nitrate transporter could somewhat enhance plants’ capacity for nitrate uptake and assimilation, particularly under nitrogen eutrophication where plant’s endogenous iHATS tends to be suppressed by high levels of nitrate. Plant growth responds to soil environmental cues such as the availability of minerals and nutrients. For example, root growth is generally enhanced by nutrient-poor conditions and inhibited by nutrient-rich conditions (Ericsson 1995). Root architecture is affected by nitrate transporters, such as the repression of lateral root initiation by NRT2.1 in Arabidopsis (Little et al. 2005). Therefore, lateral roots and primary roots may respond differently to NO_3_^−^ ion as a signaling molecule (Zhang and Forde 2000; Little et al. 2005). We observed an enhanced primary root growth, particularly in our ZmN plants, when Ca^2+^ was low but K^+^ was high (Fig. 1), suggesting that Ca^2+^ and K^+^ may be just as important as nitrate for root development. On the other hand, other nitrate and nitrite transporters such as Nrt1.1 are shown to be important for K^+^ translocation in Arabidopsis (Drechsler et al. 2015), reflecting an overlapping role for these genes and a complex network of NO_3_^−^/Ca^2+^/K^+^ interactions. Any phenotypic changes observed under different nitrate nutrient conditions must be the results of interactions among these nitrogen-responsive genes, including ZmNrt2.1 in the ZmN plants.

It is somewhat puzzling that overexpression of Nrt2.1 does not seem to significantly change NO_3_^−^ contents in plants regardless of nitrate provision, as reported by Fraisier et al. (2000) and observed by us (Additional file 1: Figure S6). It is possible that in our study expression of ZmNrt2.1 may not be functionally compatible with the native NtNAR2, therefore limiting its nitrate transportation activity. However, Fraisier et al. (2000) overexpressed a copy of native NpNrt2.1 gene in *Nicotiana plumbaginifolia* plants, therefore Nrt2.1-NAR2 compatibility is not an issue. Lack of clear cause-effect association between Nrt2.1 expression and cellular NO_3_^−^ contents under various NO_3_^−^ availabilities reflects the complex regulation of nitrate homeostasis and nitrogen metabolism during plant growth and development.

It should be pointed out that our study only examined the young plants (up to 3 weeks old) grown in the nutrient-defined medium. It remains to assess how our transgenic plants would compare to the wild type plants when grown under nitrate-challenging (too much or too little) conditions in soil throughout a life cycle. Nonetheless, our study demonstrates a feasibility that elevated high-affinity nitrate transporter transcription that does not respond to the typical suppression by high nitrate levels could be used as a strategy for enhanced nitrate sequestration by plants from the nitrate-polluting environment.

It is also worth noting that in our study, and in literature (e.g. Fraisier et al. 2000; Quaggiotti et al. 2003; Okamoto et al. 2003; Wirth et al. 2007), 1 mM was used as “low” nitrate treatment, which appears to exceed the NRT2’s operation limit of < 1 mM (Crawford and Glass 1998). In fact, due to plant growth, the nitrate content in the medium decreases rapidly. For example, in our experiments, the nitrate level on the 1 mM medium dropped below 0.5 mM after 1 week and was near 0.1–0.2 mM after 3 weeks (data not shown), with relatively healthy and reasonable-sized plants. Therefore, 1 mM nitrate should be an acceptable treatment for moderately low nitrate in nitrate transporter studies.

Calcium is a well-known important signal for a wide variety of physiological and biochemical processes in the cell (Dodd et al. 2010; Carafoli and Krebs 2016), as is an essential element for plant growth. However, its importance for nitrate signaling and nitrate-responsive gene regulation has not been fully understood or appreciated. Our results demonstrated a Ca^2+^ requirement for several nitrate-responsive genes, so that their transcription in shoots could be severely inhibited by inhibitors for calcium channel or Ca^2+^ level-regulating PLC, particularly when nitrate was limiting (Figs. 4 and 5). Thus, it is likely that Ca^2+^ and NO_3_^−^, perhaps K^+^ as well, are all important part of the transcriptional regulation network for many nitrate-responsive genes. Overall, our studies are consistent with the notion that Ca^2+^ acts as a second messenger, beside NO_3_^−^, in the nitrate signaling pathway (Riveras et al. 2015; Undurraga et al. 2017). Regardless of the role of Ca^2+^ or K^+^, nitrate availability seems to be a primary and essential signal for the cascade of gene expression for nitrogen nutrition in plants.

Using a combination of transgenic plants, inhibitors and nutrient variation, we were able to begin to dissecting, to a certain extent, the effect of a maize nitrate transporter gene on the expression of several native nitrate-responsive genes. The ZmNrt2.1 is moderately similar to the tobacco NtNrt2.1 with a similar codon usage (Additional file 1: Figure S2 and Table S3). It is not known how post-transcriptional and/or post-translational processing occurs on NRT2.1 protein in either maize or tobacco plants, as reported to take place for the Arabidopsis NRT2.1 (Wirth et al. 2007; Laugier et al. 2012). It is not clear how much, if any, ZmNrt2.1 could oligomerize with NtNAR2 to form an active nitrate transporter complex, as suggested for other plants (Tong et al. 2005; Yong et al. 2010; Pii et al. 2016). It will be important to investigate the genetic or biochemical mechanisms of how the ZmNrt2.1 interacts (or interferes) with the tobacco nitrate transporters and other native nitrate-involving genes/proteins in the host plant grown under various NO_3_^−^ or K^+^ conditions. However, regardless of whether ZmNrt2.1 was present as a monomer or functionally complexed with an accessory partner, this study clearly showed that compared to the untransformed plants, the ZmNrt2.1-expressing plants exhibited a different response in gene transcription when nitrate was limited or the Ca^2+^ signal system was impaired. For example, the fact that ZmN plants were less sensitive to LaCl_3_ inhibition for BT2 and TGA1 transcription than the wild type (Figs. 3 and 5) suggests that being part of the host’s genetic system, the maize ZmNrt2.1 somehow altered or interfered with the endogenous gene regulatory network for nitrogen nutrition in the host plant. Further exploration of this type of ectopic effects imposed by a foreign gene may be worthwhile not only for better understanding plants’ nitrogen homeostasis, but also for creating desirable plants suitable for various nitrate environments.

In summary, the transgenic tobacco plants expressing the maize nitrate transporter gene ZmNrt2.1 exhibited altered expression patterns of a set of genes that are modulated not only by nitrate, but also by calcium. Introduction of selective foreign nitrate transporter genes into crop plants, along with managing Ca^2+^ or K^+^ availability in soil, may prove to be a feasible approach to improving nitrogen use efficiency and nitrate uptake capacity, and dealing with environmental eutrophication caused by fertilizer overuse.

## Additional file

**Additional file 1: Table S1.** Sequences of primers used in this study. **Table S2.** Medium composition. **Table S3.** Comparison of codon usage. **Figure S1.** Structure of the vector p35S-ZmNrt used for tobacco transformation. **Figure S2.** Comparison of the amino acid sequences of the maize high-affinity nitrate transporter ZmNrt2.1 and the tobacco high-affinity nitrate transporter NtNrt2.1. **Figure S3.** PCR analysis of genomic DNA extracted from leaves of wild type and putative transgenic tobacco plants. **Figure S4.** RT-PCR screening for transgenic plants. **Figure S5.** Gene expression analysis. **Figure S6.** Soluble nitrate contents in plant shoots and roots. **Figure S7.** Representative photographs of plant growth with various levels of nitrate, Ca^2+^ and K^+^. **Figure S8.** NtNAR gene expression analysis.
