# Supplementary material for: Tobacco plants expressing the maize nitrate transporter ZmNrt2.1 exhibit altered responses of growth and gene expression to nitrate and calcium
Source: Bot Stud. 2017 Nov 15;58:51. doi: 10.1186/s40529-017-0203-9 (PMC5688054; doi:10.1186/s40529-017-0203-9)
Supplement: Supplementary file 1 — Additional file 1: Table S1. Sequences of primers used in this study. Table S2. Medium composition. Table S3. Comparison of codon usage. Figure S1. Structure of the vector p35S-ZmNrt used for tobacco transformation. Figure S2. Comparison of the amino acid sequences of the maize high-affinity nitrate transporter ZmNrt2.1 and the tobacco high-affinity nitrate transporter NtNrt2.1. Figure S3. PCR analysis of genomic DNA extracted from leaves of wild type and putative transgenic tobacco plants. Figure S4. RT-PCR screening for transgenic plants. Figure S5. Gene expression analysis. Figure S6. Soluble nitrate contents in plant shoots and roots. Figure S7. Representative photographs of plant growth with various levels of nitrate, Ca2+ and K+. Figure S8. NtNAR gene expression analysis. [file 40529_2017_203_MOESM1_ESM.docx]

**Additional Information**

**Tobacco plants expressing the maize nitrate transporter ZmNrt2.1 exhibit altered responses of growth and gene expression to nitrate and calcium**

Abubakar Ibrahim^1,2^, Xiao-Lu Jin^1^, Yu-Bin Zhang^1,3^, Jessica Cruz^1^, Paveena Vichyavichien^1^, Nwadiuto Esiobu^1^, Xing-Hai Zhang^1,^*

^1^ Department of Biological Sciences, Florida Atlantic University, Boca Raton, FL 33431, USA

^2^ Department of Soil Sciences, Modibbo Adama University of Technology, Yola, Nigeria

^3^ Center for Marine Resources and Environments, Guangdong Ocean University, Zhanjiang, China

* To whom correspondence should be addressed. E-mail: xhzhang@fau.edu

Table S1 Sequences of primers used in this study

| Name Sequence (5’→ 3’) Usage |
| --- |
| NtN-1 AAGCTTCACACGGCTGGAAT PCR for tobacco high-affinity Nrt2.1  NtN-2 GGCGTTACATTAGGCGGAGT |
| NtN-3 CCAAGCCTACGACCACCAAA PCR for tobacco low-affinity Nrt1.1  NtN-4 TGGCTGCCTTGTCCAAGAAT |
| NtN-5 GCAGGATGGGGAAATATGGG qPCR for tobacco high-affinity Nrt2.1 and 2.2  NtN-6 GGCAAATCTTGGCCAAGAGT |
| NtN-7 GGTTATGGAATATGTGCWTGTGC qPCR for tobacco low-affinity Nrt1.1 (4 isoforms)  NtN-8 CTGAAGCAATTTGTGTCAATGGA |
| NtAR-1 TGGATATCTGCTCCATTGTCTTC qPCR for tobacco NAR2.1  NtAR-2 TGACTTGCCTTAGCCTTTCTC |
| ZmN-1 GAGCAATGGTGGCAAGTTCG PCR for *Zea mays* high-affinity Nrt2.1  ZmN-2 AACCTGACGGTGATGTAGCC |
| ZmN-3 GCCATGGTGCTCTTCTCCTT PCR for *Zea mays* high-affinity Nrt2.1  ZmN-4 TTCCACTCGGAGGCGTAGTA |
| ZmN-1 GAGCAATGGTGGCAAGTTCG qPCR for *Zea mays* high-affinity Nrt2.1  ZmN-5 GATCCAGGGAGGTGGAAGGTG |
| ZmN-1 GAGCAATGGTGGCAAGTTCG qPCR for *Zea mays* high-affinity Nrt2.1  ZmN-6 CACGGAGAAGAGACGGACGC |
| ZmN-7 GCATCATGTCCGACATGGGC qPCR for *Zea mays* high-affinity Nrt2.1  ZmN-8 CGCCGGCGGTCTGGAGA |
| EF1-f CTTGGTGGTATTGACAAGCGTGT qPCR for tobacco elongation factor 1α  EF1-r TGTCAAGCACCCAGGCATACT |
| NtL-1 AGTTACATTCCACCGACCTAAGACT qPCR for tobacco ribosomal protein L25  NtL-2 AATCTTCTTCATTGCAGACTCTGTGG |
| TGA-1 CAGATACATCGGCAACCTTGAT qPCR for tobacco TGACG sequence-specific binding  TGA-2 AATAGGTTTACGTGCTTCTGGT protein1 |
| TGA-3 TCAGCTGAGCGATTCTTCTTAT qPCR for tobacco TGACG sequence-specific binding  TGA-4 GAGTTGTTGTTCTGTCAATGGC protein1 |
| NtBT1 CCACAGCTGAAGCAGAGATGCA qPCR for tobacco Bric-a-Brac/Tramtrack/Broad gene 2  NtBT2 GCAAGTTGAAGCACATCCACTGC |
| NtBT3 TCAAGAAAGTTGAGGAGACTGAGG qPCR for tobacco Bric-a-Brac/Tramtrack/Broad gene 2  NtBT4 TCAGCCTCATCCATGAACTGTA |
| NtBT5 TACTTCTCAGAACAGAGAAGTTACCT qPCR for tobacco Bric-a-Brac/Tramtrack/Broad gene 2  NtBT6 GCCTCTACAGCAGGACATATGA |

Table S2 Medium composition

| Medium Type | Nutrient composition |
| --- | --- |
| 1 mM N - 5 mM Ca - 5 mM K | 1 mM NO_3_^-^, 1 mM PO_4_^-2^, 5 mM K^+^, 2 mM MgSO_4_, 5 mM Ca^+2^, 0.1 mM Fe^+2^, 13 mM Cl^-^, trace elements, pH 6.0 |
| 1 mM N - 1 mM Ca – 5 mM K | 1 mM NO_3_^-^, 1 mM PO_4_^-2^, 5 mM K^+^, 2 mM MgSO_4_, 1 mM Ca^+2^, 0.1 mM Fe^+2^, 5 mM Cl^-^, trace elements, pH 6.0 |
| 1 mM N - 1 mM Ca - 50 mM K | 1 mM NO_3_^-^, 1 mM PO_4_^-2^, 50 mM K^+^, 2 mM MgSO_4_, 1 mM Ca^+2^, 0.1 mM Fe^+2^, 46 mM Cl^-^, trace elements, pH 6.0 |
| 1 mM N – 5 mM Ca - 1 mM K | 1 mM NO_3_^-^, 1 mM PO_4_^-2^, 1 mM K^+^, 2 mM MgSO_4_, 5 mM Ca^+2^, 0.1 mM Fe^+2^, 9 mM Cl^-^, trace elements, pH 6.0 |
| 10 mM N - 5 mM Ca - 5 mM K | 10 mM NO_3_^-^, 1 mM PO_4_^-2^, 5 mM K^+^, 2 mM MgSO_4_, 5 mM Ca^+2^, 0.1 mM Fe^+2^, 4 mM Cl^-^, trace elements, pH 6.0 |
| 10 mM N-1 mM Ca - 5 mM K | 10 mM NO_3_^-^, 1 mM PO_4_^-2^, 5 mM K^+^, 2 mM MgSO_4_, 1 mM Ca^+2^, 0.1 mM Fe^+2^, 9 μM Cl^-^, trace elements, pH 6.0 |
| 10 mM N - 1 mM Ca - 50 mM K | 10 mM NO_3_^-^, 1 mM PO_4_^-2^, 50 mM K^+^, 2 mM MgSO_4_, 1 mM Ca^+2^, 0.1 mM Fe^+2^, 41 mM Cl^-^, trace elements, pH 6.0 |
| 10 mM N – 5 mM Ca - 1 mM K | 10 mM NO_3_^-^, 1 mM PO_4_^-2^, 1 mM K^+^, 2 mM MgSO_4_, 5 mM Ca^+2^, 0.1 mM Fe^+2^, 9 μM Cl^-^, trace elements, pH 6.0 |
| Murashige & Skoög (MS) | 39.4 mM NO_3_^-^, 20.6 mM NH_4_^+^, 1.2 mM PO_4_^-2^, 20 mM K^+^, 1.5 mM MgSO_4_, 3 mM Ca^+2^, 0.1 mM Fe^+2^, 6 μM Cl^-^, trace elements, pH 5.8-6.0 |

Table S3 Comparison of codon usage

**AmAcid Codon Fraction**

**NtNrt2.1 NtNrt2.2 ZmNrt2.1**

Ala GCG 0.09 0.07 0.25

Ala GCA 0.36 0.24 0.05

Ala GCT 0.34 0.57 0.07

Ala GCC 0.20 0.13 0.63

Cys TGT 0.50 0.78 0.00

Cys TGC 0.50 0.22 1.00

Asp GAT 0.76 0.81 0.20

Asp GAC 0.24 0.19 0.80

Glu GAG 0.62 0.46 1.00

Glu GAA 0.38 0.54 0.00

Phe TTT 0.38 0.57 0.11

Phe TTC 0.62 0.42 0.89

Gly GGG 0.10 0.06 0.17

Gly GGA 0.45 0.48 0.08

Gly GGT 0.31 0.39 0.02

Gly GGC 0.14 0.07 0.73

His CAT 0.63 0.60 0.27

His CAC 0.38 0.40 0.73

Ile ATA 0.24 0.14 0.00

Ile ATT 0.59 0.70 0.04

Ile ATC 0.18 0.16 0.96

Lys AAG 0.50 0.52 1.00

Lys AAA 0.50 0.48 0.00

Leu TTG 0.20 0.21 0.00

Leu TTA 0.22 0.23 0.00

Leu CTG 0.13 0.02 0.32

Leu CTA 0.09 0.09 0.00

Leu CTT 0.31 0.34 0.00

Leu CTC 0.04 0.11 0.68

Met ATG 1.00 1.00 1.00

Asn AAT 0.59 0.53 0.06

Asn AAC 0.41 0.47 0.94

Pro CCG 0.04 0.13 0.45

Pro CCA 0.59 0.29 0.10

Pro CCT 0.33 0.58 0.05

Pro CCC 0.04 0.00 0.40

Gln CAG 0.33 0.23 1.00

Gln CAA 0.67 0.77 0.00

Arg AGG 0.17 0.11 0.15

Arg AGA 0.39 0.53 0.00

Arg CGG 0.04 0.05 0.04

Arg CGA 0.09 0.11 0.04

Arg CGT 0.22 0.21 0.07

Arg CGC 0.09 0.00 0.70

Ser AGT 0.14 0.16 0.00

Ser AGC 0.05 0.02 0.31

Ser TCG 0.17 0.12 0.12

Ser TCA 0.26 0.23 0.05

Ser TCT 0.24 0.37 0.02

Ser TCC 0.14 0.09 0.50

Thr ACG 0.11 0.13 0.33

Thr ACA 0.30 0.41 0.03

Thr ACT 0.49 0.38 0.00

Thr ACC 0.11 0.08 0.63

Val GTG 0.26 0.19 0.30

Val GTA 0.12 0.09 0.00

Val GTT 0.41 0.63 0.03

Val GTC 0.21 0.09 0.68

Trp TGG 1.00 1.00 1.00

Tyr TAT 0.50 0.46 0.00

Tyr TAC 0.50 0.54 1.00

End TGA 1.00 1.00 0.00

End TAG 0.00 0.00 0.00

End TAA 0.00 0.00 1.00

**
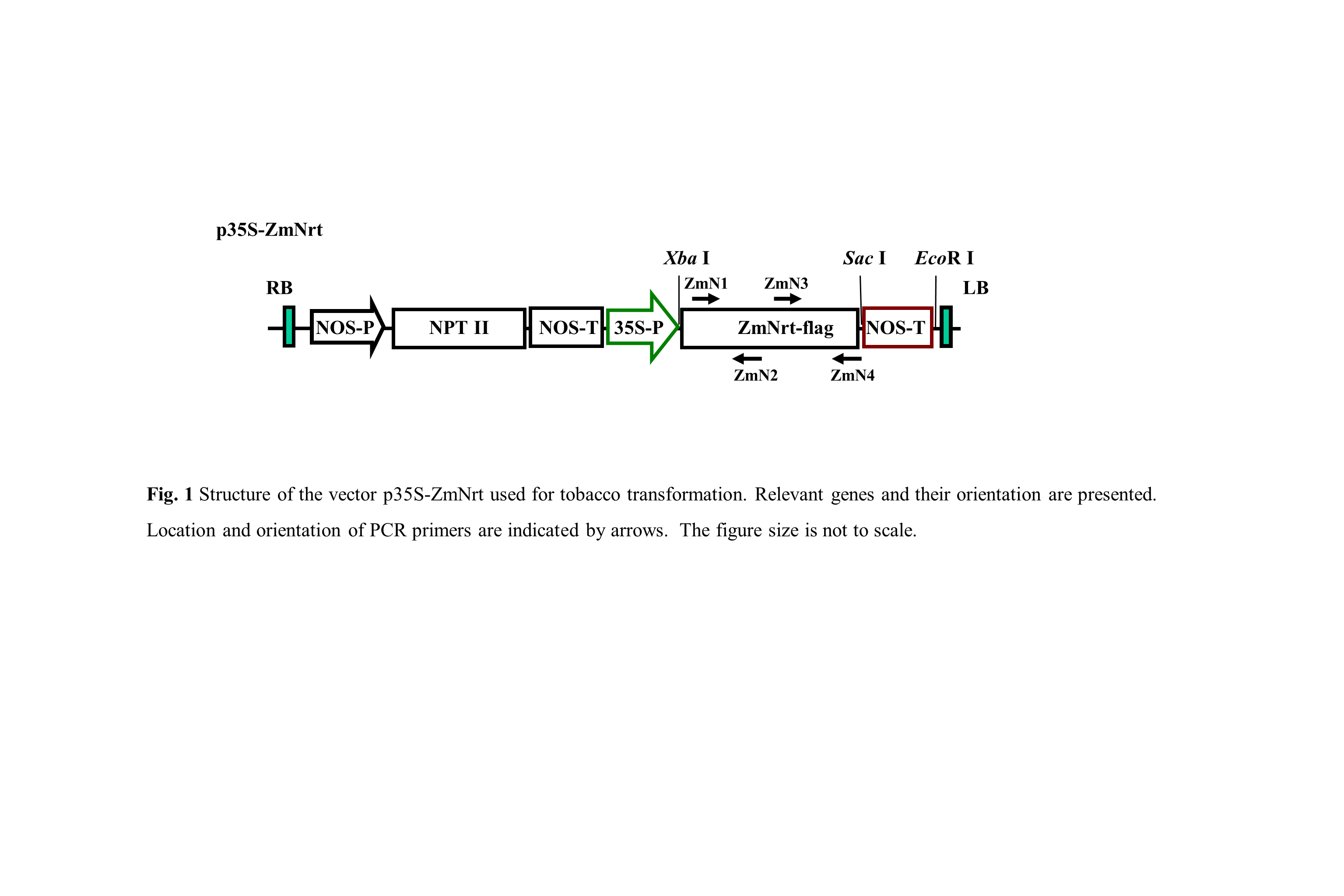
**

**Fig. S1**. Structure of the vector p35S-ZmNrt used for tobacco transformation. Relevant genes and their orientation are presented. Location and orientation of PCR primers are indicated by arrows. The figure size is not to scale.

ZmNrt2.1 MAAV-GAPGSSLHGVTGREPAFAFSTEHEEAASNG-GKFDLPVDSEHKAKSVRLFSVANP

NtNrt2.1 MGDIEGEPGSSMHGVTGREPVLAFSVASPMVPTDTTAKFSVPVDTEHKAKIFKFYSFSKP

*. : * ****:********.:***. ..:: .**.:***:***** .:::*.::*

ZmNrt2.1 HMRTFHLSWISFFTCFVSTFAAAPLVPIIRDNLNLTKADIGNAGVASVSGSIFSRLTMGA

NtNrt2.1 HGLTFQLSWISFFTCFVSTFAAAPLVPIIRDNLNLTKMDVGNAGVASVSGSILSRLVMGA

* **:******************************* *:************:***.***

ZmNrt2.1 VCDLLGPRYGCAFLIMLSAPTVFCMSLIDDAAGYITVRFLIGFSLATFVSCQYWMSTMFS

NtNrt2.1 VCDMLGPRYGCAFLIMLSAPTVFCMSFVSSAGGYVAVRFMIGFSLATFVSCQYWMSTMFN

***:**********************::..*.**::***:*******************.

ZmNrt2.1 SKIIGTVNGLAAGWGTMGRRRHAAHMPLVYDVIRKCGATPFTAWRLAYFVPGLMHVVMGV

NtNrt2.1 SQIIGLVNGTAAGWGNMGGGATQLIMPIVYDIIRRAGATPFTAWRIAFFIPGWLHIVMGI

*:*** *** *****.** **:***:**:.*********:*:*:** :*:***:

ZmNrt2.1 LVLTLGQDLPDGNLRSLQKKGNVNKDSFSKVMWYAVINYRTWIFVLLYGYCMGVELTTDN

NtNrt2.1 LVLTLGQDLPDGNRGDLQKKGDVSKDKFSNILWYAATNYRTWIFVLLYGYSMGVELSTDN

************* .*****:*.**.**:::***. *************.*****:***

ZmNrt2.1 VIAEYMYDRFDLDLRVAGTIAACFGMANIVARPMGGIMSDMGARYWGMRARLWNIWILQT

NtNrt2.1 VIAEYFFDRFDLKLHTAGIIAATFGMANLLARPFGGFSSDYAAKRFGMRGRLWVLWILQT

*****::*****.*:.** *** *****::***:**: ** .*: :***.*** :*****

ZmNrt2.1 AGGAFCLWLGRASTLPVSVVAMVLFSFCAQAACGAIFGVIPFVSRRSLGIISGMTGAGGN

NtNrt2.1 LGGVFCVLLGRSNPLPIAVTFMILFSIGAQAACGATFGIIPFISRRSLGIISGMTGAGGN

**.**: ***:..**::*. *:***: ******* **:***:*****************

ZmNrt2.1 FGAGLTQLLFFTSSTYSTGRGLEYMGIMIMACTLPVVFVHFPQWGSMFFPPS---ATADE

NtNrt2.1 FGSGLTQLLFFTSSKYSTATGLTYMGLMIIGCTLPVTFCHFPQWGSMFFPPTKDPVKGSE

**:***********.***. ** ***:**:.*****.* ************: ....*

**FLAG**

ZmNrt2.1 EGYYASEWNDDEKSKGLHSASLKFAENSRSERGKRN-VIQADAAATPEHVDYKDDDDK

NtNrt2.1 EHYYAAEYTEAERQKGMHQNSLKFAENCRSERGKRVGSAPTPPNLTPNRV

* ***:*:.: *:.**:*. *******.******* : . **::*

**Fig. S2**. Comparison of the amino acid sequences of the maize high-affinity nitrate transporter ZmNrt2.1 and the tobacco high-affinity nitrate transporter NtNrt2.1. Identical residues (*), strongly similar residues (:) and weakly similar residues (.) are shown. The FLAG tag (underlined) was fused to the C-terminus of ZmNrt2.1 to facilitate Western blot analysis.

**
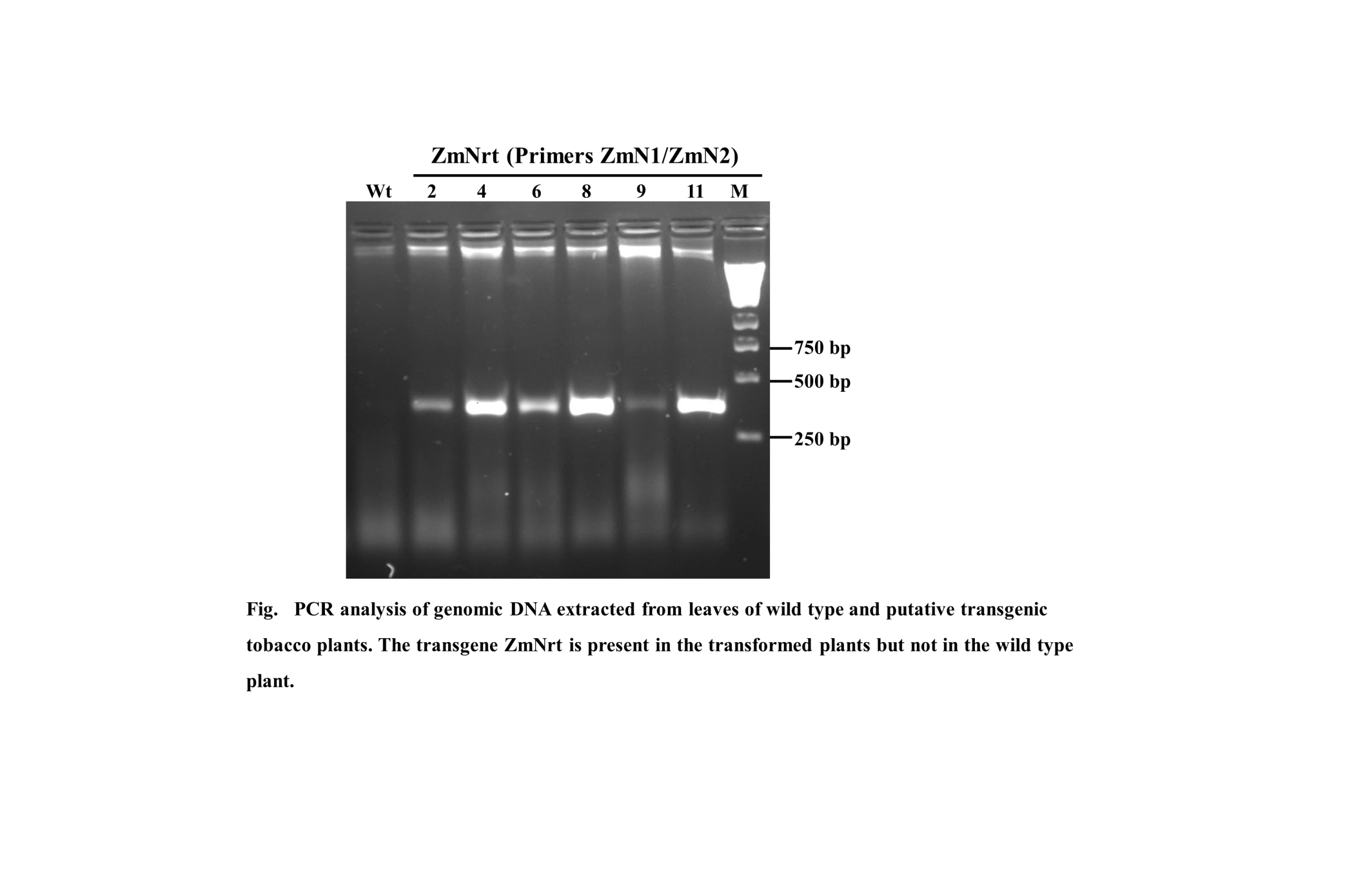
**

**Fig. S3.** PCR analysis of genomic DNA extracted from leaves of wild type and putative transgenic tobacco plants. The transgene ZmNrt2.1 was present in the transformed plants but not in the wild type (Wt) plant.


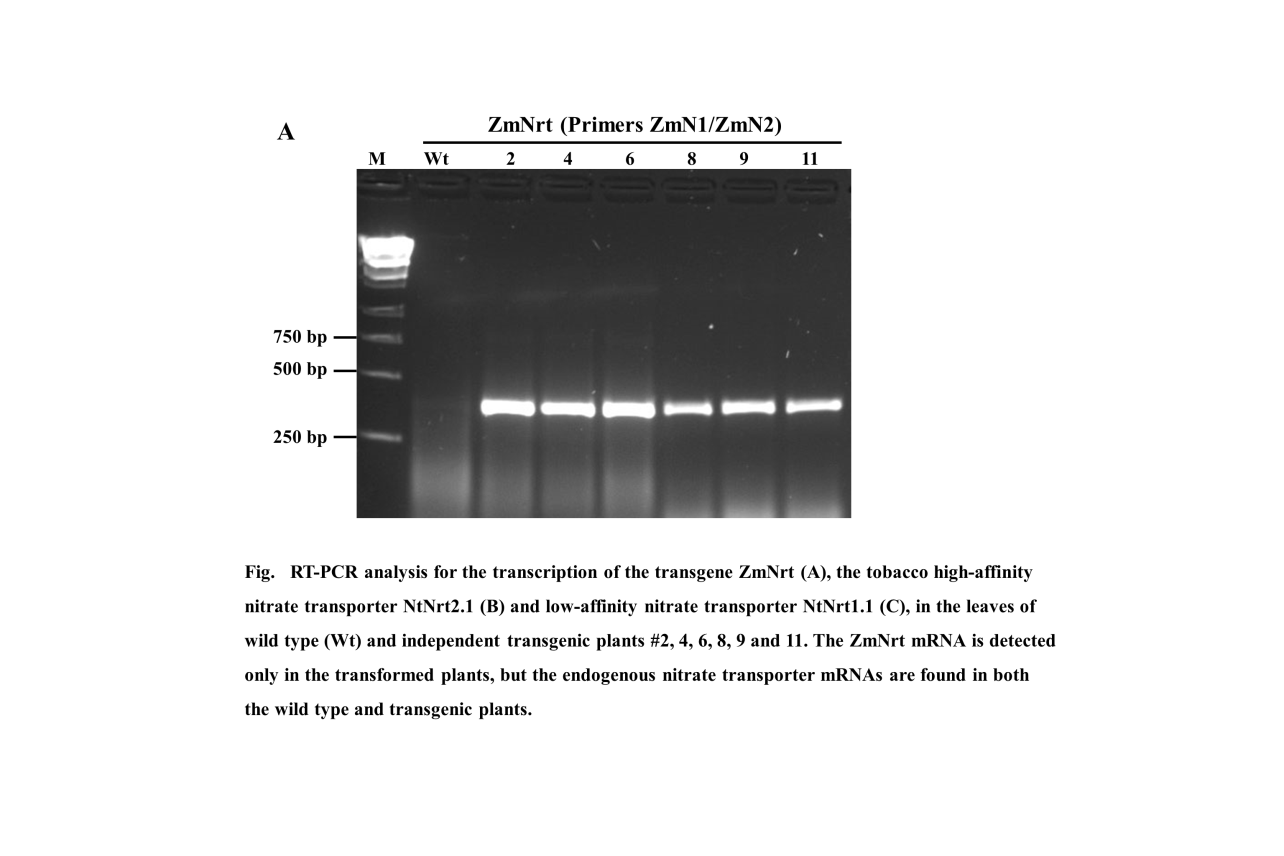

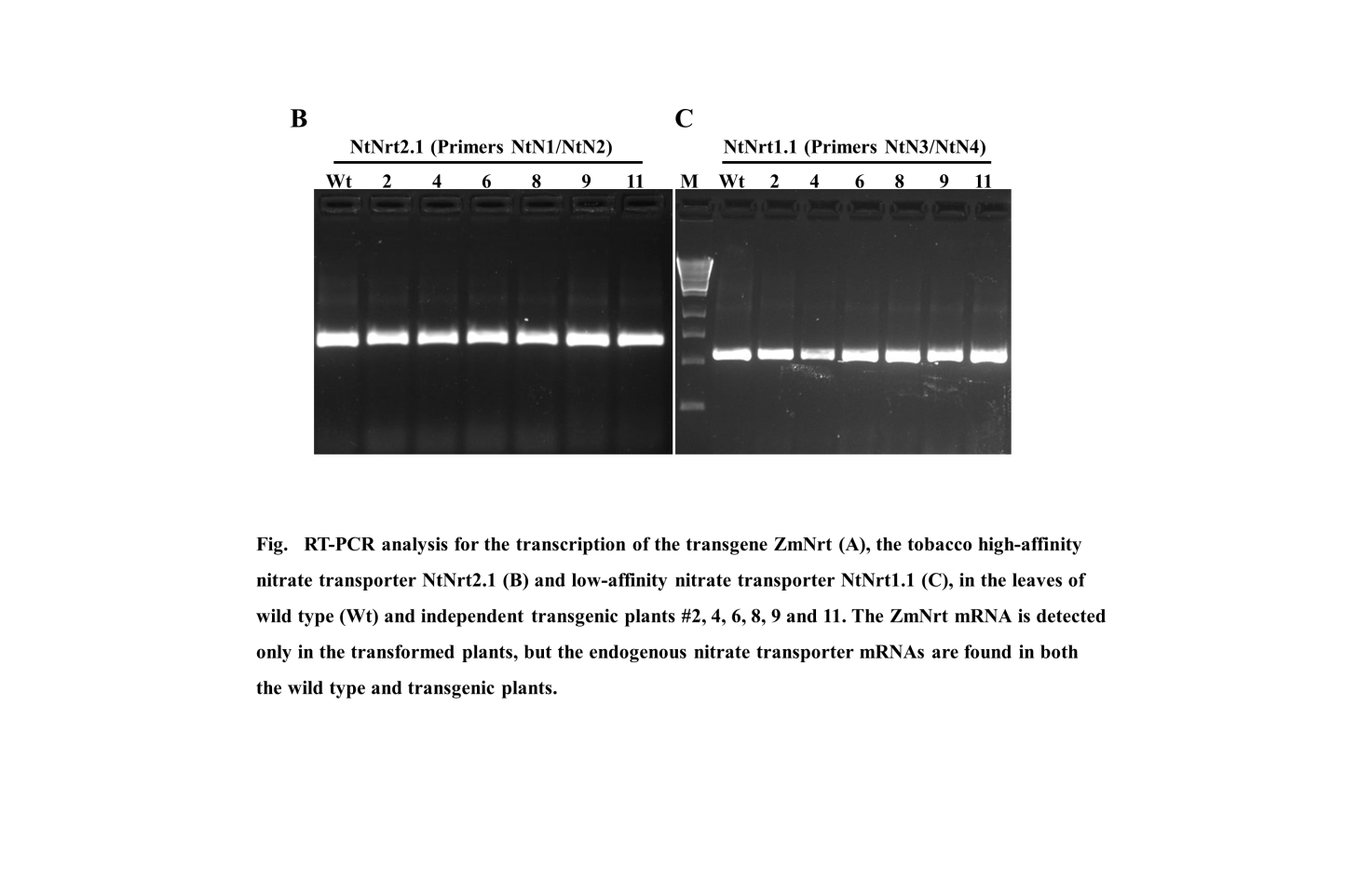


**Fig. S4.** RT-PCR screening for transgenic plants. RT-PCR was used to detect transcription of the transgene ZmNrt2.1 (A), the tobacco high-affinity nitrate transporter NtNrt2.1 (B) and low-affinity nitrate transporter NtNrt1.1 (C), in the leaves of wild type (Wt) and independent transgenic plant lines ZmN#2, 4, 6, 8, 9 and 11. ZmNrt2.1 mRNA was detected only in the transformed plants, but the endogenous nitrate transporter mRNAs were found in both the wild type and the transgenic plants.

**
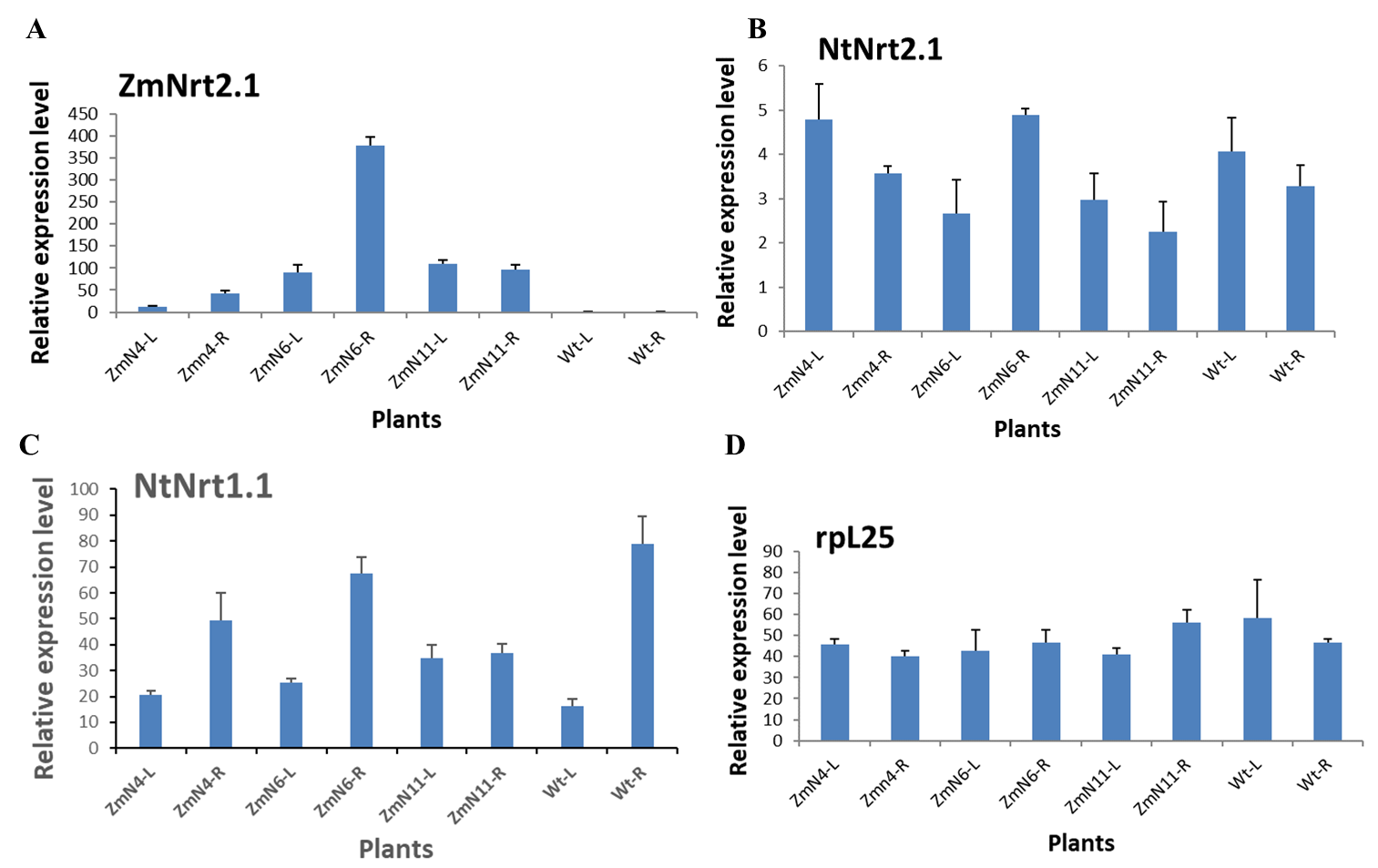
**

**
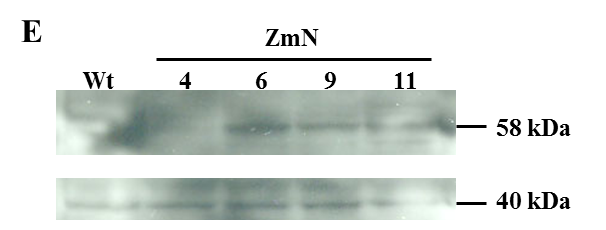
**

**Fig. S5.** Gene expression analysis. Relative transcription levels for the transgene ZmNrt2.1 (A), tobacco endogenous high-affinity nitrate transporter NtNrt2.1 (B), tobacco low-affinity nitrate transporter NtNrt1.1 (C), and tobacco ribosomal protein L25 (D) from plants grown in MS medium for 3 weeks were assayed by RT-qPCR. Western blot analysis detected the presence of the 58 kDa FLAG-tagged ZmNrt2.1 protein in the transgenic plants by anti-FLAG antibody (E). The 40 kDa protein that cross-reacted with the antibody was used as internal standard to indicate protein loading. ZmN 4, 6, 11: independent transgenic lines; Wt: wild type; L: leaf, R: root.

**
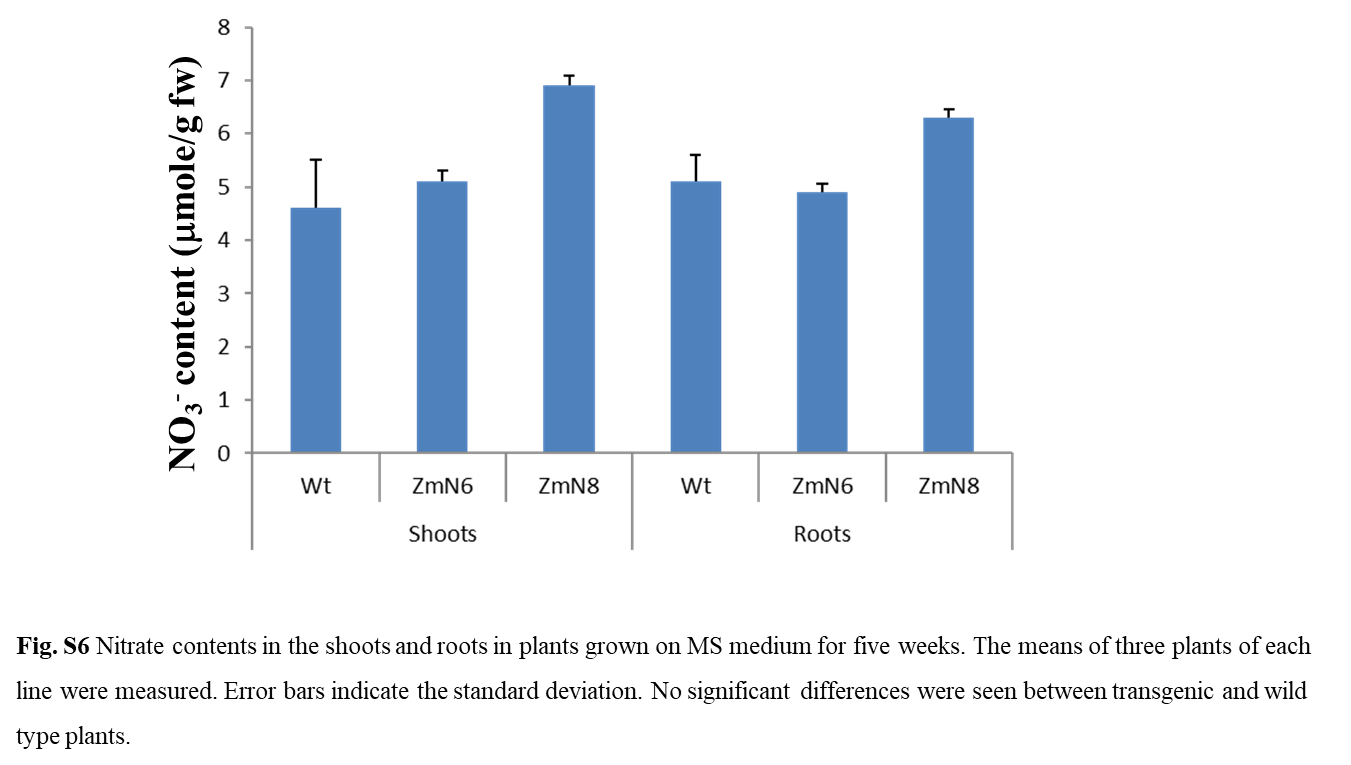
**

**Fig. S6.** Soluble nitrate contents in plant shoots and roots. Three plants of each line grown on MS medium for five weeks were measured. Error bars indicate the standard deviation. No significant differences were seen between transgenic and wild type plants.

**
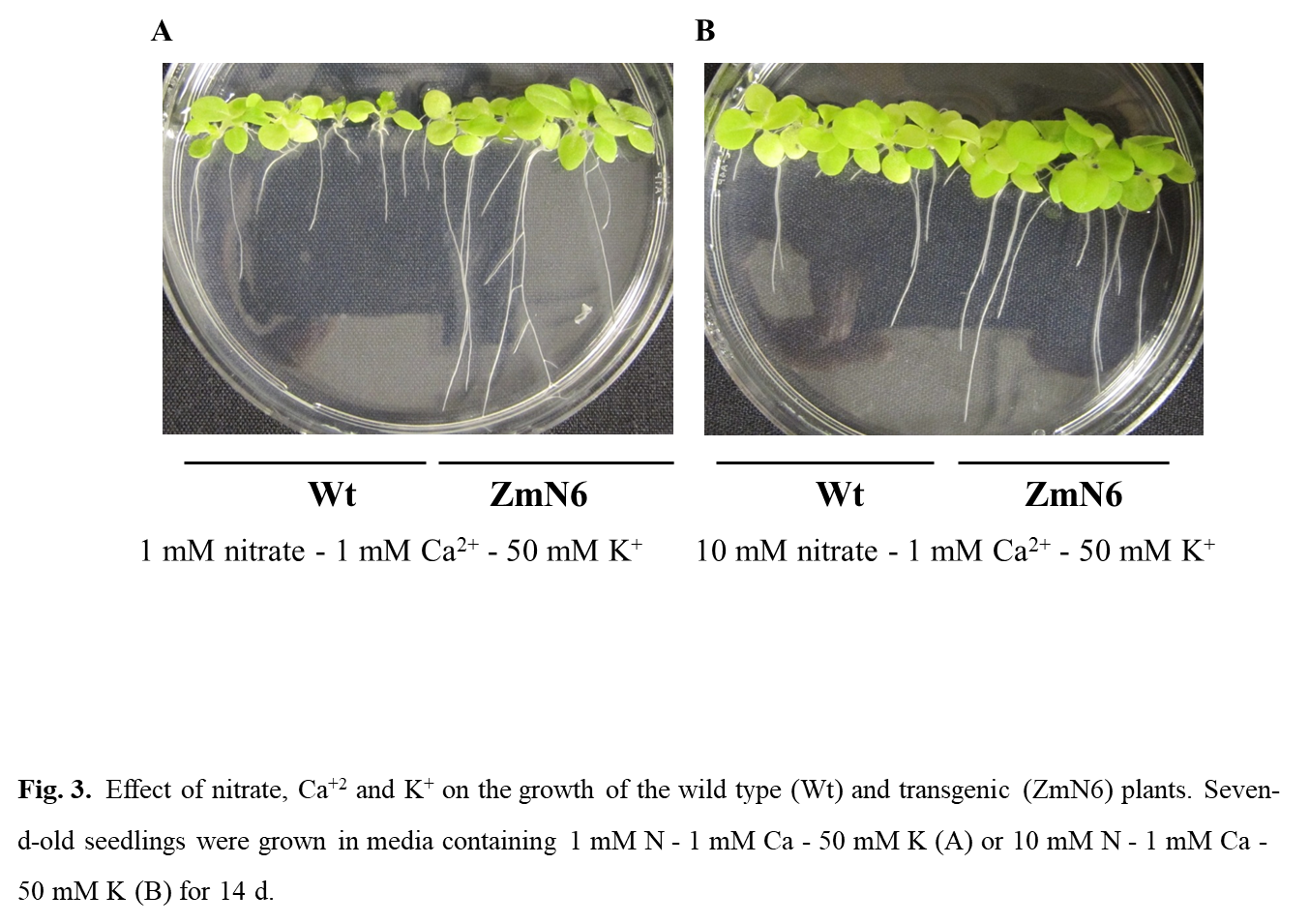

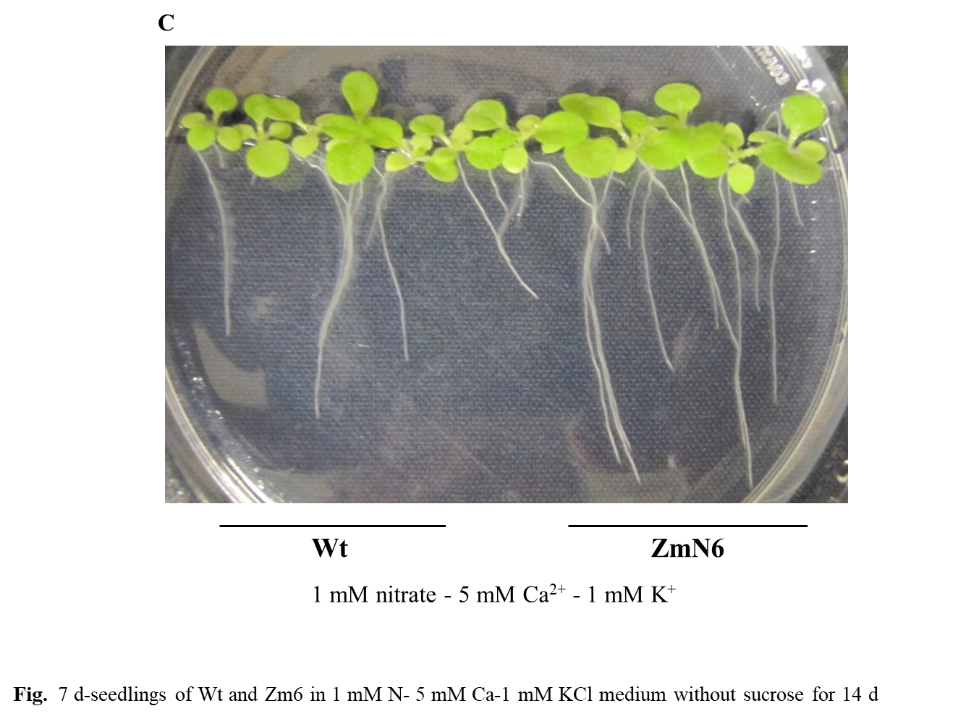

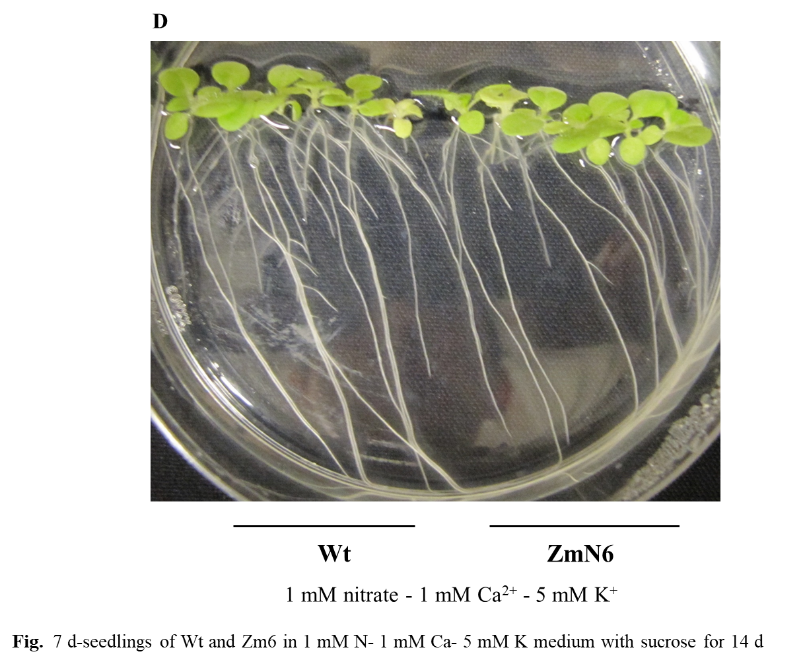

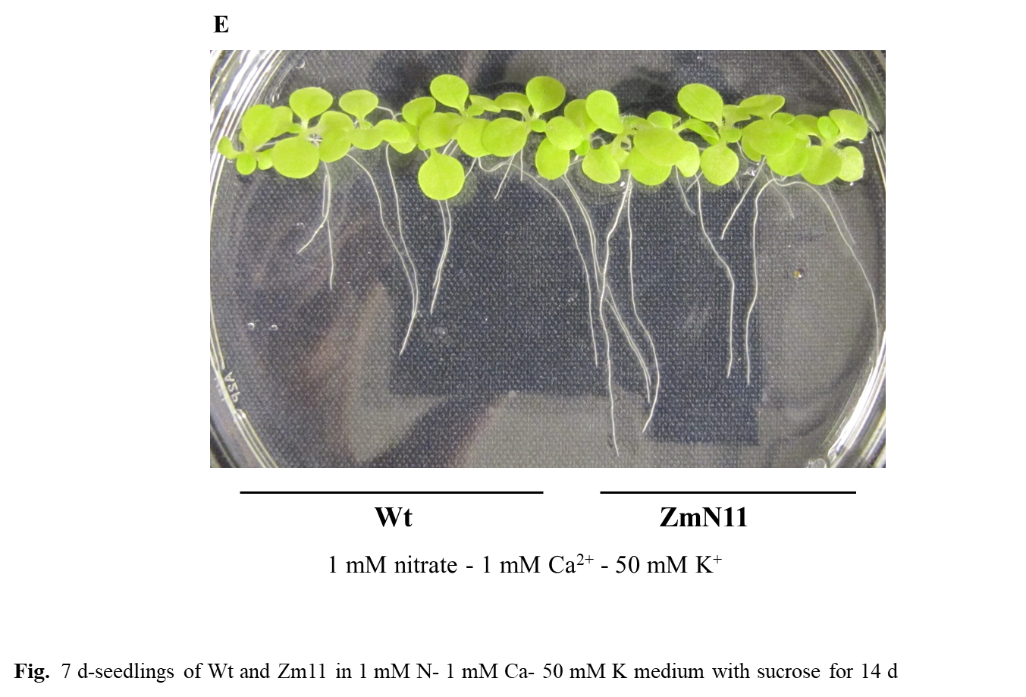

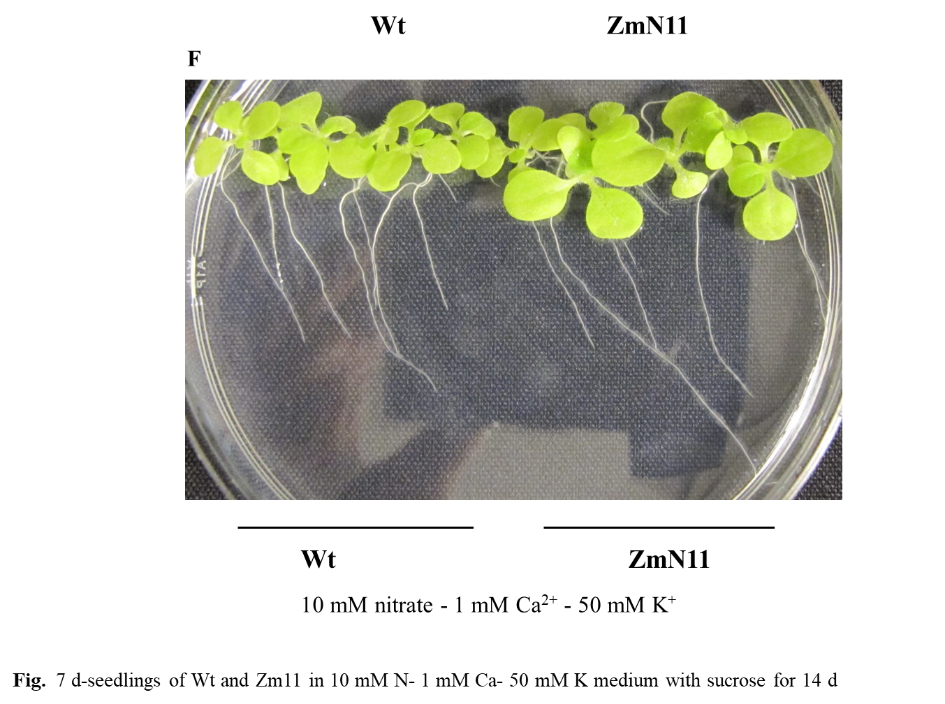
**

**
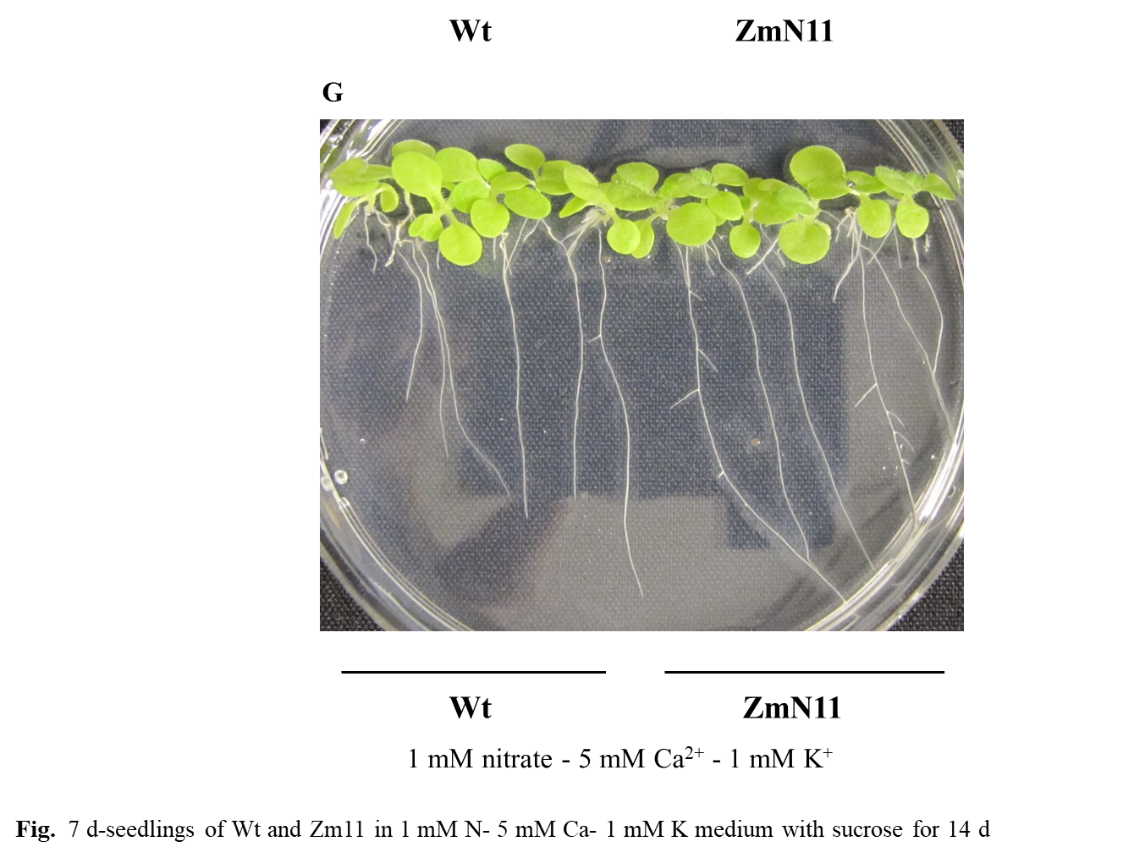

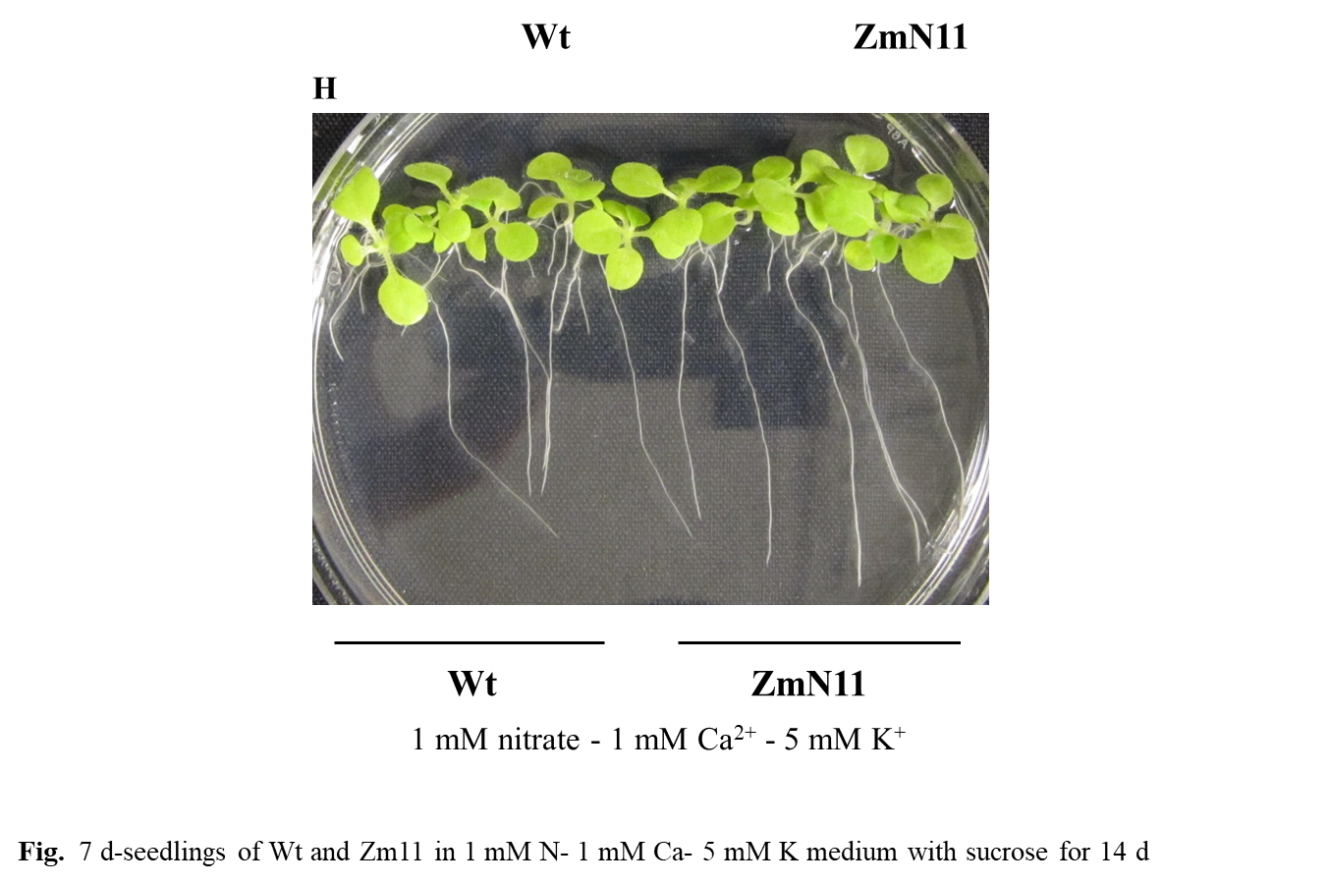
**

**Fig. S7.** Representative photographs of plant growth with various levels of nitrate, Ca^2+^ and K^+^. Seven-d-old seedlings of the wild type (Wt) and transgenic plants ZmN6 and ZmN11 were grown in various media for 14 d.


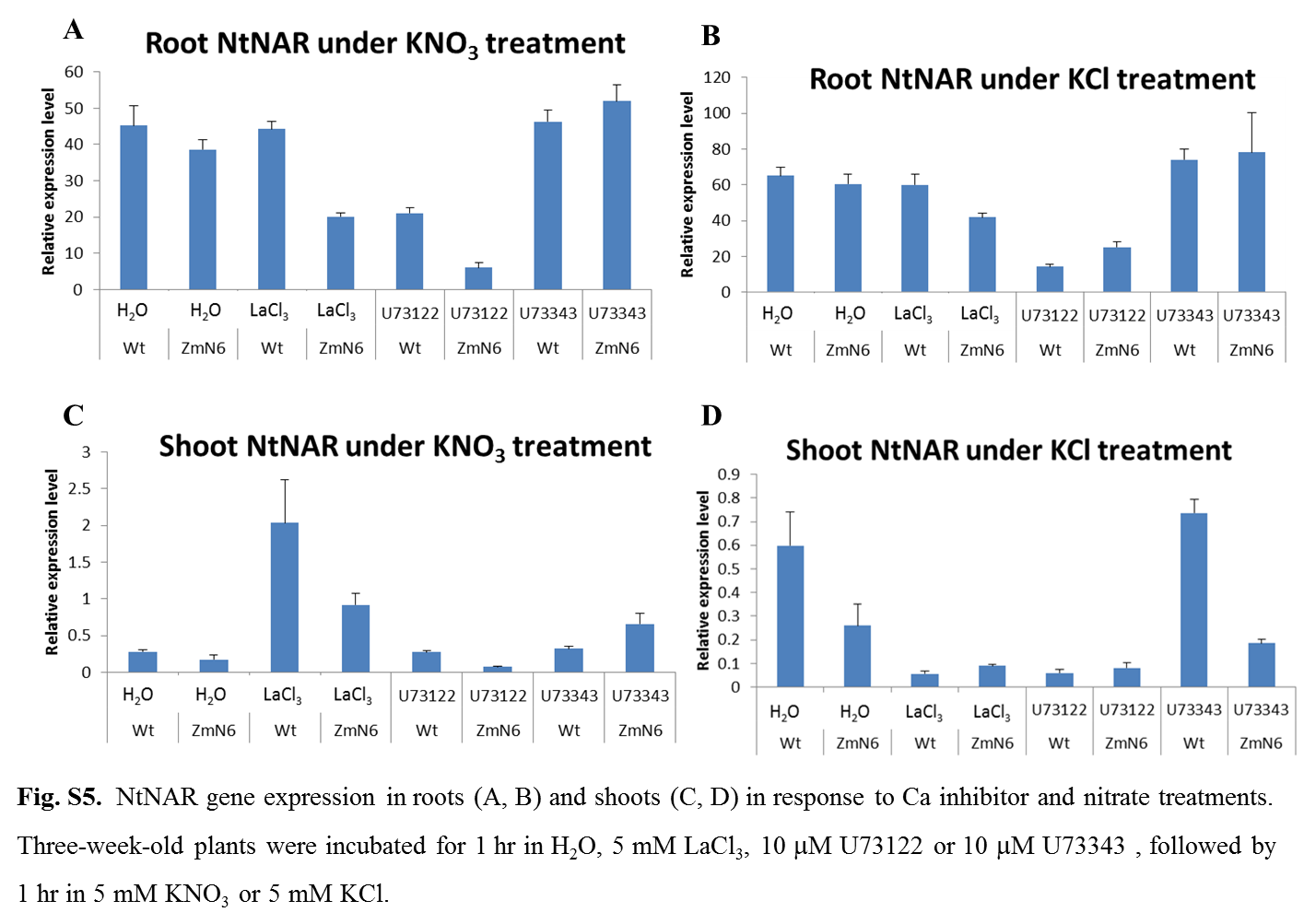


**Fig. S8.** NtNAR gene expression analysis. Relative transcription levels of NtNAR in roots (A, B) and shoots (C, D) in response to Ca inhibitor and nitrate treatments were measured. Three-week-old plants were incubated for 1 hr in H_2_O, 5 mM LaCl_3_, 10 μM U73122 or 10 μM U73343 , followed by 1 hr in 5 mM KNO_3_ or 5 mM KCl. ZmN6: ZmNrt2.1 transgenic plant; Wt: wild type plant.
